# Transcriptome-Wide Analysis of Interplay between mRNA Stability, Translation and Small RNAs in Response to Neuronal Membrane Depolarization

**DOI:** 10.3390/ijms21197086

**Published:** 2020-09-25

**Authors:** Dylan J. Kiltschewskij, Murray J. Cairns

**Affiliations:** 1 School of Biomedical Sciences and Pharmacy, University of Newcastle, Callaghan 2308, Australia; dylan.kiltschewskij@uon.edu.au; 2 Centre for Brain and Mental Health Research, Hunter Medical Research Institute, New Lambton 2305, Australia; 3 Schizophrenia Research Institute, Randwick 2031, Australia

**Keywords:** translation, post-transcriptional regulation, neuron, ribosome profiling, RNA degradation, microRNA, tRNA-derived small RNA

## Abstract

Experience-dependent changes to neural circuitry are shaped by spatially-restricted activity-dependent mRNA translation. Although the complexity of mRNA translation in neuronal cells is widely appreciated, translational profiles associated with neuronal excitation remain largely uncharacterized, and the associated regulatory mechanisms are poorly understood. Here, we employed ribosome profiling, mRNA sequencing and small RNA sequencing to profile transcriptome-wide changes in mRNA translation after whole cell depolarization of differentiated neuroblast cultures, and investigate the contribution of sequence-specific regulatory mechanisms. Immediately after depolarization, a functional partition between transcriptional and translational responses was uncovered, in which many mRNAs were subjected to significant changes in abundance or ribosomal occupancy, but not both. After an extended (2 h) post-stimulus rest phase, however, these changes became synchronized, suggesting that there are different layers of post-transcriptional regulation which are temporally separated but become coordinated over time. Globally, changes in mRNA abundance and translation were found to be associated with a number of intrinsic mRNA features, including mRNA length, GC% and secondary structures; however, the effect of these factors differed between both post-depolarization time-points. Furthermore, small RNA sequencing revealed that miRNAs and tRNA-derived small RNA fragments were subjected to peak changes in expression immediately after stimulation, during which these molecules were predominantly associated with fluctuations in mRNA abundance, consistent with known regulatory mechanisms. These data suggest that excitation-associated neuronal translation is subjected to extensive temporal coordination, with substantial contributions from a number of sequence-dependent regulatory mechanisms.

## 1. Introduction

Excitation of neurons is known to induce distinct patterns of gene expression which regulate activity-dependent fine-tuning of neuronal networks [1,2,3]. Since these events are thought to underpin the molecular basis of phenomena, including learning, memory and cognition, an extensive body of studies have characterized transcriptional profiles associated with neuronal excitation in a variety of models [1,2,3]. In contrast, the dynamics of excitation-associated mRNA translation remain poorly understood, despite the known significance of translation in neuronal plasticity. Early studies investigating the effects of translational inhibitors on neuronal long-term potentiation were the first to establish the requirement for translation in the transition between short- and long-lasting forms of plasticity [4,5]. The importance of excitation-associated translation has since been further exemplified through the detection of translationally active ribosomes and mRNAs in close proximity to the synapse [6,7,8,9,10,11], as well as the visualization of activity-dependent translational profiles for a small subset of neuronal genes (reviewed in [12]). In parallel, dysregulation of translation and associated regulatory systems has emerged as a common feature amongst a number of psychiatric disorders, including schizophrenia, fragile X syndrome, major depressive disorder and bipolar disorder [13,14], underscoring a particularly important role in cognitive function and stressing the need for comprehensive profiling of translational dynamics in the neuronal context. However, while activity-dependent patterns of translation have been identified for a subset of neuronal genes [15,16,17,18,19], transcriptome-wide profiles of activity-dependent translation and associated regulatory mechanisms remain poorly characterized.

In mammalian cells, sequence-specific control of mRNA translation is conferred by a number of cis-acting and trans-acting regulatory elements. For instance, the expression of secondary structures within the mRNA 5′ untranslated region (UTR) or coding sequence (CDS) can modulate translation initiation and ribosomal scanning [20,21,22], while structural motifs can recruit RNA-binding proteins (RBP) capable of enhancing or repressing translation at multiple stages [23]. Given the unique spatiotemporal distribution of mRNA and translational machinery in neuronal cells, additional sequence-specific mechanisms are thought to provide enhanced translational fine-tuning in the neural context. One system thought to bear particular importance during neuronal activity is microRNA (miRNA), a class of ~22 nt non-coding RNAs which post-transcriptionally repress expression of complementary mRNAs [24]. In neurons, miRNAs have been shown to colocalize with mRNA and translational machinery [25,26,27,28], undergo rapid maturation near the synapse in response to activity input [28,29] and control neuronal features associated with neuronal plasticity (reviewed in [30]). However, the overall extent to which these molecules contribute to activity-dependent translational dynamics is currently unknown, as is the predominant repressive mechanism employed by activity-sensitive miRNAs.

In the present study, we conducted parallel ribosome profiling, mRNA sequencing and small RNA sequencing to investigate translational changes associated with repeated K^+^ depolarization of SH-SY5Y cells and explore the sequence-specific regulatory factors involved. We report that K^+^ depolarization induces robust modulation of mRNA translation, wherein the processing of mRNAs involved in neuron-specific functions is transiently prioritized. Interestingly, we additionally found that early translational changes are poorly associated with fluctuations in mRNA abundance; however, these variables become comparatively synchronized 2 h after the stimulation paradigm. Finally, our results indicate that intrinsic mRNA features are associated with mRNA abundance and translation in a temporally coordinated manner, whereas depolarization-sensitive miRNAs and tRNA-derived small RNA fragments were specifically associated with mRNA abundance.

## 2. Results

### 2.1. Sequencing of K^+^ Depolarised SH-SY5Y Cells

**Figure 1:**
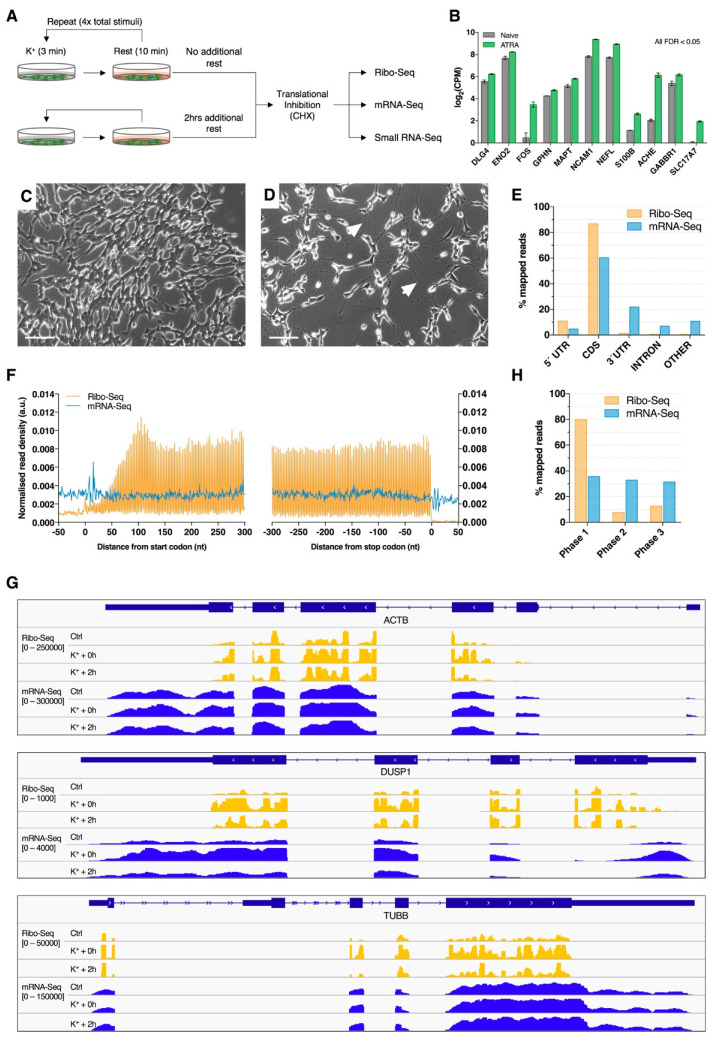
Ribosome profiling of neuronally differentiated K^+^ depolarized SH-SY5Y cells. (**A**) Schematic overview of the membrane depolarization paradigm employed in this study. (**B**) Expression of known neuronal marker genes in ATRA-treated SH-SY5Y cells (7 days) relative to naïve cells from a previous study. All genes were subjected to statistically significant upregulation (FDR < 0.05). (**C**,**D**) Phase contrast images of naïve (**C**) and ATRA-treated (**D**) SH-SY5Y cells. Note the change in cellular morphology and extensive neurite development after ATRA treatment (white arrows). Scale bar = 100 µm. (**E**) Percentage of Ribo-Seq and mRNA-Seq reads aligning to transcriptome features. Composite data are shown after merging biological replicates and treatment groups. (**F**) Metagene analysis of read density around transcript start and stop codons, showing high ribosome-protected mRNA fragment (RPF) density between coding sequence (CDS) start and stop codons. RPF reads were calibrated to estimated P-sites (see supplementary methods for further details). (**G**) Screenshots of RPF (orange) and mRNA (blue) read depth (RPKM) for representative genes: ACTB, DUSP1 and TUBB. (**H**) Sub-codon phasing of RPF and mRNA reads, revealing that RPFs exhibit a preference for the first canonical reading frame, whereas mRNA-Seq data displays comparatively even distribution across all three frames.

To investigate patterns of mRNA translation associated with membrane depolarization, differentiated SH-SY5Y cells were repeatedly stimulated with K^+^ and subjected to parallel ribosome profiling (Ribo-Seq) and mRNA sequencing (mRNA-Seq; Figure 1A). Successful neuronal differentiation was assessed by analyzing cellular morphology during ATRA treatment, which was later supported by the upregulation of known neuronal marker genes relative to naïve cells (Figure 1B–D). Ribo-Seq and mRNA-Seq respectively produced 62.11 × 10^6^ (± 31.37 × 10^6^) and 53.03 × 10^6^ (± 31.36 × 10^6^) raw reads, of which 14.76 × 10^6^ (± 9.75 × 10^6^) and 48.18 × 10^6^ (± 28.35 × 10^6^) were retained for further analysis after quality control and genome mapping (Table S1). Overall, mRNA-Seq libraries predominantly aligned to the CDS (~60%) and 3’UTR (~22%) and were evenly distributed across start/stop codons and reading frames as a result of random fragmentation (Figure 1E–H). In contrast, Ribo-Seq libraries preferentially aligned to mRNA coding sequences (CDS; ~87%) and 5′UTRs (~11%), as expected for translationally-active ribosomes (Figure 1E). Metagene analysis of read density around CDS start and stop codons additionally revealed that the Ribo-Seq reads were predominantly distributed between these features, which was supported by visual inspection of read coverage for specific genes (Figure 1F,G). Furthermore, sub-codon phasing uncovered a clear bias for CDS reading frame 1 (~80% of reads), demonstrating the triplet periodicity of ribosomal translocation (Figure 1H). These observations therefore support the successful enrichment of ribosome-protected mRNA fragments (RPFs).

### 2.2. Differential mRNA Translation Associated with K^+^ Depolarisation

**Figure 2:**
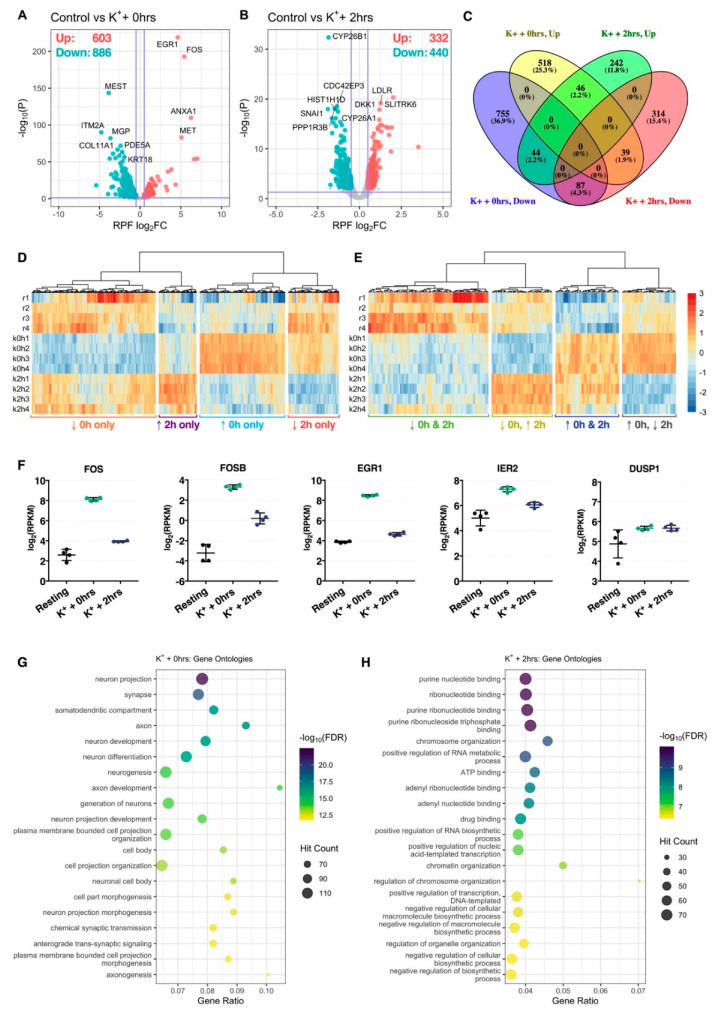
Translational changes induced by membrane depolarization. (**A**,**B**) Volcano plots comparing translational log_2_ fold change (log_2_FC) and –log_10_ *p*-values of genes immediately (**A**) or 2 h (**B**) after depolarization, relative to resting cells. Significantly upregulated and downregulated genes are marked in red and blue, respectively, with Benjamini–Hochberg FDR < 0.05 and log_2_FC > |± 0.5| considered significant. Horizontal line represents nominal *p* < 0.05. (**C**) Venn diagram depicting the overlap of significant genes between both time-points. (**D**) Heat map comparing RPF RPKM across all samples for genes significantly regulated at one time-point only. Each cell corresponds to the RPKM standard deviation relative to the row-wise mean, with red corresponding to high expression, and blue to low expression (scale bar shared with (**E**)). (**E**) As in (**D**), except depicting genes significantly regulated at both time-points. (**F**) Expression of known immediate-early genes, FOS, FOSB, EGR1, IER2 and DUSP1, at the RPF level. All five genes were significantly upregulated at both time-points. (**G**,**H**) Bubble plots showing top 20 significantly enriched gene ontology terms after analysis of genes significantly upregulated immediately (**G**) or 2 h (**H**) after stimulation. Note that “Gene Ratio” refers to the overlap between the query gene set versus the number of genes categorized under the gene ontology term.

Differential expression analysis identified 1489 genes (603 upregulated, 886 downregulated) significantly regulated at the RPF level immediately after repeated K^+^ stimulation, while 772 genes (332 upregulated, 440 downregulated) were additionally identified after subjecting cells to an extended 2-h rest phase post-stimulus (Figure 2A,B, Tables S2 and S3). Comparison of significant genes between both time-points revealed a strong temporal partition, reflected in the identification of 1829 (89.5%) genes exhibiting translational responses in one condition only (Figure 2C,D). Of the remaining 216 (10.6%) genes, 46 (2.2%) and 87 (4.3%) were persistently upregulated or downregulated, respectively, while 83 (4.1%) exhibited opposite patterns of expression between both time-points (Figure 2C,E). Amongst genes with persistently increased ribosomal occupancy, we identified representation of known neuronal immediate-early genes FOSB, FOS, EGR1, IER2 and DUSP1, supporting the efficacy of the K^+^ depolarisation paradigm (Figure 2F).

GO term analysis of significant genes uncovered a clear functional division between both post-stimulus time-points. Immediately after depolarization, translationally prioritized genes were considerably enriched for neuron-specific GO annotations, with top terms including neuron projection, synapse, somatodendritic compartment and axon, amongst others (Figure 2G). A number of terms with relevance to neuronal excitation were also uncovered, such as chemical synaptic transmission and anterograde trans-synaptic signaling (Figure 2G, Table S4). These annotations were diminished 2 h after depolarization, wherein top enriched terms predominantly related to nucleotide binding, including purine nucleotide binding, ribonucleotide binding and adenyl ribonucleotide binding, and transcription-associated biological processes, such as positive regulation of nucleic acid-templated transcription, positive regulation of RNA biosynthetic process and chromatin organization (Figure 2H, Table S5).

### 2.3. Disparity Between mRNA Abundance and Translation

**Figure 3:**
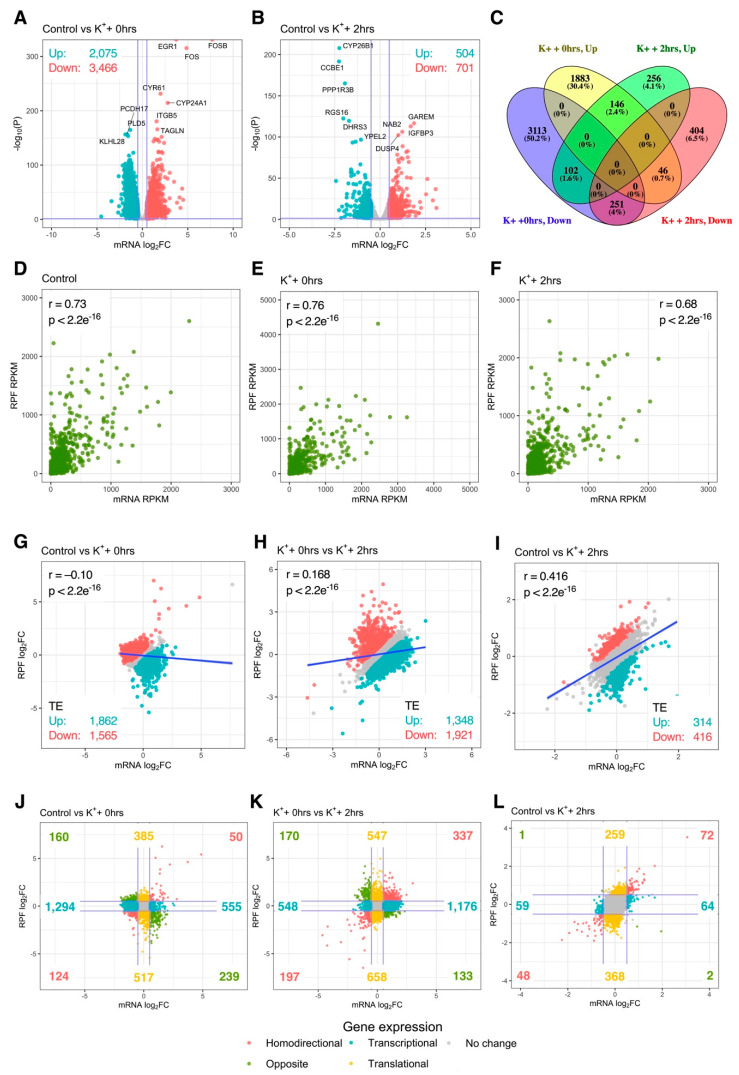
Interplay between mRNA abundance and translational activity. (**A**,**B**) Volcano plots comparing mRNA-Seq log_2_ fold change (log_2_FC) and –log_10_ *p*-values of genes immediately (**A**) or 2 h (**B**) after depolarization, relative to resting cells. Significantly upregulated and downregulated genes are marked in red and blue, respectively, with Benjamini–Hochberg FDR < 0.05 and log_2_FC > |± 0.5| considered significant. Horizontal line represents nominal *p* < 0.05. (**C**) Venn diagram depicting the overlap of significant genes between both time-points. (**D**–**F**) Gene-wise comparison of mRNA-Seq and Ribo-Seq RPKM in all experimental groups. Pearson’s correlation coefficient and associated *p*-values are reported (top left/right). (**G**–**I**) Gene-wise comparison of mRNA-Seq and Ribo-Seq log_2_FC. Pearson’s correlation coefficient and associated *p*-values are reported (top left). Genes with significantly increased or decreased translational efficiency (TE) are marked in red and blue, respectively. Dark blue line represents fitted linear model. (**J**–**L**) As in (**G**–**I**), except after categorizing genes based on expression at the mRNA and RPF levels. Blue lines represent log_2_FC = ± 0.5. “Homodirectional” refers to genes significantly altered at both the mRNA and RPF levels in the same direction; “Opposite” refers to genes significantly changed at both levels, but in opposite directions; “Transcriptional” refers to genes significantly altered at the mRNA level only; “Translational” refers to genes significantly changes at the RPF level only.

To examine the relationship between mRNA abundance and translational activity, depolarization-associated remodeling of mRNA levels was next quantified via mRNA-Seq. Differential analysis at the mRNA level firstly revealed 5541 (2075 upregulated, 3466 downregulated) and 1205 (504 upregulated, 701 downregulated) significantly altered genes immediately and 2 h after stimulation, respectively, indicating that remodeling of mRNA abundance was extensive (Figure 3A,B). Proportionally, overlap of significantly regulated genes between conditions was comparable to the RPF-level, with 5656 (91.2%) genes exhibiting temporal specificity, while 545 (8.7%) were persistently changed at both time-points (Figure 3C). Overall, the large changes in mRNA abundance and translation were consistent with differential expression of genes involved in these processes (Figure S4).

We next analyzed the extent of transcriptional vs. translational regulation after K^+^ stimulation. When all biological groups were analyzed individually, a strong correlation between mRNA and RPF counts was identified in both treated and untreated cells (Pearson’s r ≥ 0.86, *p* < 2.2e^−16^; Figure 3D–F). However, when mRNA and RPF fold changes (FC) were compared, poor correlation was observed immediately after the K^+^ paradigm (r = −0.10, *p* < 2.2e^−16^), suggesting early depolarization-associated changes in mRNA abundance and translation exhibited limited concordance (Figure 3G). Interestingly, these factors became positively associated during the experimental time course (r_0 hrs vs. 2 hrs_ = 0.17, *p* < 2.2e^−16^; r_Ctrl vs. 2 hrs_ = 0.42, *p* < 2.2e^−16^), such that changes in mRNA and RPF expression became increasingly synchronized (Figure 3H,I). These findings were supported by analysis of translational efficiency (TE), which found 3427 (1862 upregulated, 1565 downregulated) genes with disproportionate changes in mRNA and RPF levels immediately after K^+^ stimulation, whereas only 730 (314 upregulated, 416 downregulated) were significant after 2 h (Figure 3G,I). Overall, these results indicate that early changes in depolarization-induced translational activity are poorly associated with changes in mRNA abundance.

### 2.4. Extensive Post-Transcriptional Buffering of Translation Immediately After Depolarisation

To further dissect the inconsistency between mRNA and RPF expression changes, we explored the magnitude of regulation at both levels for all quantified genes. Immediately after K^+^ stimulation, only 174 genes exhibited significant mRNA and RPF changes in the same direction (homodirectional changes), consistent with the poor correlation between mRNA and RPF log_2_FC (Figure 3J). In contrast, 1849 genes were regulated exclusively at the mRNA level, suggesting post-transcriptional buffering of mRNA translation was prominent at this time-point. While pure translational regulation was additionally uncovered for a subset of 902 genes, this affected less than half the number of genes subjected to post-transcriptional buffering. Interestingly, a clear functional partition between transcriptional and translational responses was also identified, in which genes subjected to post-transcriptional buffering were predominantly enriched for GO terms relating to translation and the cell cycle, whereas genes associated with neuron-specific functions, extracellular matrix interactions and cellular junctions were subjected to pure translational regulation (Table S6). Overall, these changes were similar upon comparison of both time-points (Figure 3K). Furthermore, at the 2-h time-point, a higher proportion of genes were regulated at the RPF level only (627) compared to the mRNA level only (123), implying a decline in the post-transcriptional buffering of translation (Figure 3L). Together, these findings suggest that early responses to depolarization were associated with pervasive post-transcriptional buffering of translation which largely subsided 2 h post-stimulus.

### 2.5. Post-Transcriptional Dynamics are Associated with Intrinsic Transcript Features

Since early responses to K^+^ depolarization were associated with complex patterns of mRNA modulation, we next sought to explore sequence-specific regulatory mechanisms contributing to these post-transcriptional dynamics. We therefore examined the relationship between transcriptome-wide changes in mRNA expression and six transcript regulatory elements previously shown to influence mRNA levels and/or translatability, specifically sequence length, GC%, minimum free energy of secondary structures (normalized to sequence length; NMFE), g-quadruplex (G4) structures, upstream AUG (uAUG) codons and rare codon frequency (RCF). Where applicable, we analyzed the effects of these regulatory elements in the 5′UTR, CDS and 3′UTR separately.

**Figure 4:**
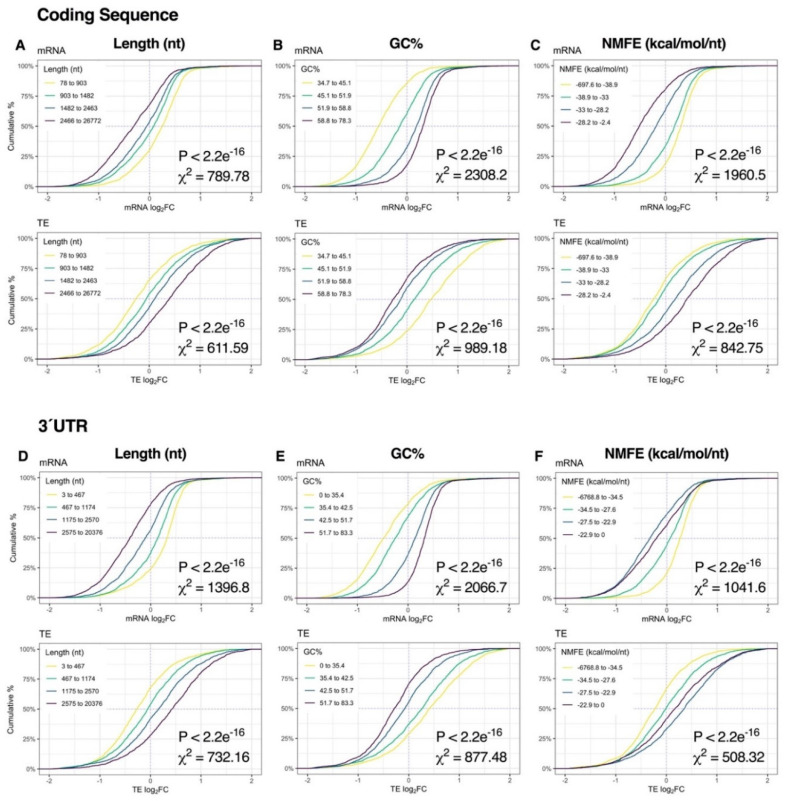
Relationship between mRNA dynamics and sequence features immediately after depolarization. (**A**–**C**) Cumulative density plots depicting changes in mRNA and translational efficiency (TE) log_2_FC after binning genes into quartiles by coding sequence length (**A**), GC% (**B**) or minimum free energy of secondary structures normalized to sequence length (NMFE) (**C**). All groups were compared via Kruskal–Wallis test, with associated *p*-values and Chi-squared (χ^2^) test statistics reported (bottom right). (**D**–**F**) As in (**A**–**C**), except examining the same features in the 3′ untranslated region (UTR). See Supplementary Figure S5 for the full version with RPF data, Supplementary Figure S6 for 5′UTR data and Supplementary Figure S8 for G4, uAUG and rare codon frequency (RCF) data.

Immediately after K^+^ stimulation, binning of genes into quartiles by sequence length, GC% or NMFE resulted in robust stratification of mRNA expression, with the strongest responses observed in the CDS and 3′UTR (*p* < 2.2e^−16^, χ^2^ ≥ 508.32; Figure 4, Figure S5). More specifically, sequence length and NMFE were inversely proportional to mRNA log_2_FC, whereas GC% was proportional. We also observed a strong association between mRNA abundance, 5′UTR length and 5′UTR GC% (*p* < 2.2e^−16^, χ^2^ ≥ 178,66), suggesting sequence features in the 5′UTR also contribute to regulation of depolarization-associated mRNA expression (Figure S6). Surprisingly, RPF profiles exhibited comparatively no response (Figures S5 and S6 ), and consequently, patterns of TE log_2_FC were similar to the mRNA level, albeit in opposite directions (Figure 4, Figures S5 and S6 ).

**Figure 5:**
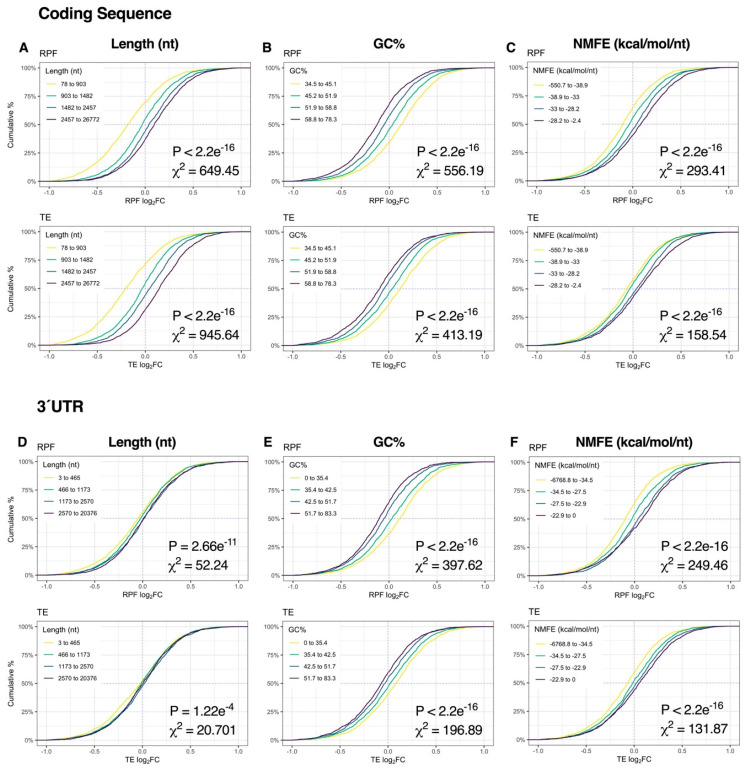
Relationship between mRNA dynamics and sequence features 2 h after depolarization. (**A–C**) Cumulative density plots depicting changes in RPF and TE log_2_FC after binning genes into quartiles by coding sequence length (**A**), GC% (**B**) or minimum free energy of secondary structures normalized to sequence length (NMFE) (**C**). All groups were compared via Kruskal–Wallis test, with associated *p*-values and Chi-squared (χ^2^) test statistics reported (bottom right). (**D**–**F**) As in (**A**–**C**), except examining the same features in the 3′UTR. See Supplementary Figure S7 for the full version with RPF data, Supplementary Figure S6 for 5′UTR data and Supplementary Figure S9 for G4, uAUG and RCF data.

At the 2-h time-point, sequence length, GC% and NMFE were more strongly associated with changes in RPF expression than mRNA levels, reflecting the transition from post-transcriptional buffering to proportionally higher translational regulation (Figure 3J). Specifically, CDS length and CDS/3′UTR NMFE were robustly proportional to RPF log_2_FC, whereas CDS/3′UTR GC% was inversely proportional (all *p* < 2.2e^−16^, χ^2^ ≥ 131.87; Figure 5A–F, Figure S7). In contrast, the magnitude of the effect was considerably lower upon analysis of 5′UTR features (*p* ≥ 2.77e^−11^, χ^2^ ≤ 52.16; Figure S6). Given the overall modest responses at the mRNA level, the resultant TE distribution profiles were generally similar to the RPF level. Together, these results suggest that mRNA length, GC% and secondary structures are initially associated with mRNA abundance immediately after repeated K^+^ stimulation but become more associated with mRNA translation after 2 h.

Changes in mRNA dynamics associated with G4 structures were comparatively modest. Immediately after depolarization, an increased number of G4 structures in the CDS was associated with increased mRNA (*p* < 2.2e^−16^ χ^2^ = 96.06) and RPF expression (*p* = 1.22e^−8^, χ^2^ = 39.9; Figure S8). In addition, 5′UTR G4 content was associated with increased TE (*p* = 3.69e^−7^, χ^2^ = 32.72; Figure S8). However, no clear patterns of G4-associated expression were identified after 2 h (Figure S9). Likewise, the number of uAUG codons was inversely proportional to mRNA expression (*p* < 2.2e^−16^, χ^2^ = 242.02) and proportional to TE (*p* < 2.2e^−16^, χ^2^ = 184.01) immediately after excitation, but exhibited no response at 2 h (Figures S8 and S9). Furthermore, RCF exhibited no clear direction of effect at either time-point (Figures S8 and S9).

### 2.6. Depolarisation-Sensitive MicroRNAs are Associated with Modulation of mRNA Abundance

**Figure 6:**
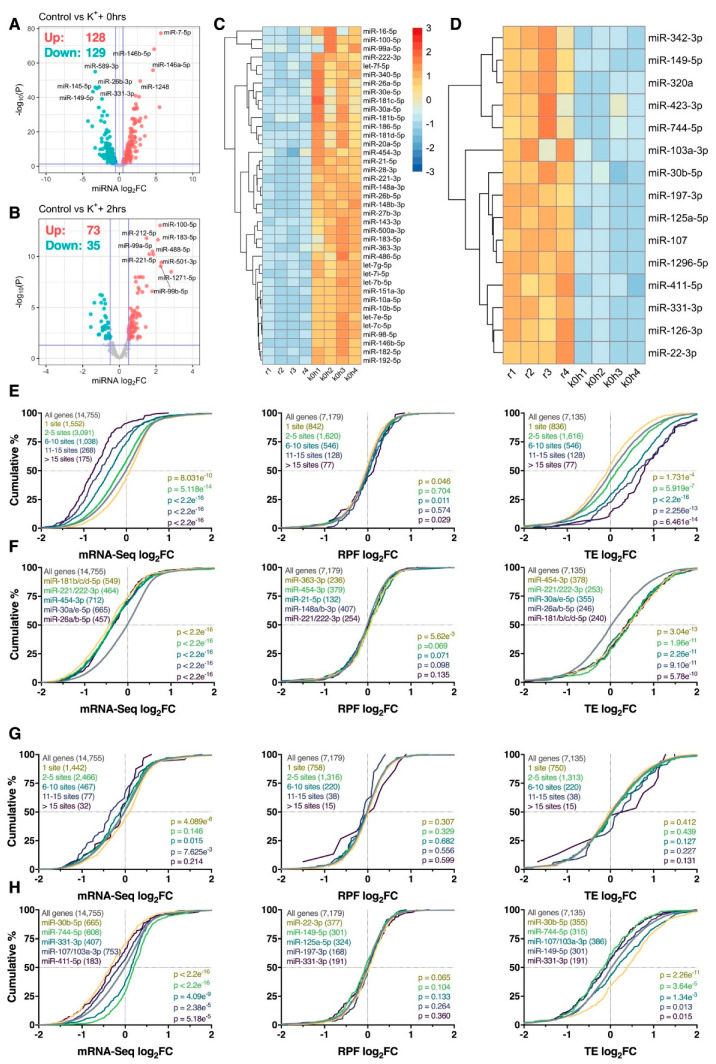
Neuronally enriched miRNAs are associated with changes in mRNA abundance. (**A**,**B**) Volcano plots comparing log_2_ fold change (log_2_FC) and –log_10_ *p*-values of mature miRNAs immediately (**A**) or 2 h (**B**) after depolarization, relative to resting cells. Significantly upregulated and downregulated miRNAs are marked in red and blue, respectively, with Benjamini–Hochberg FDR < 0.05 and log_2_fc > |± 0.5| considered significant. Horizontal line represents nominal *p* < 0.05. (**C**) Heat map depicting expression of 39 miRNAs significantly upregulated immediately after the depolarization paradigm and within the top 20% of brain-expressed miRNAs as reported by the miRMine database [31]. Each cell corresponds to the counts per million (CPM) standard deviation relative to the row-wise mean, with red corresponding to high expression, and blue to low expression. (**D**) As in (**C**), except depicting the expression of 15 significantly downregulated and brain-enriched miRNAs. Note that panels (**C**) and (**D**) share the same scale. (**E**) Cumulative density plots of mRNAs collectively targeted by the miRNAs are presented in panel (**C**), stratified by the number of expressed miRNA binding sites. Groups were compared to the entire transcriptome via two-sided Kolmogorov–Smirnov test, with *p*-values (and number of genes analyzed) reported (bottom right). (**F**) As in (**E**), except after analyzing the target genes of each miRNA individually. Top 5 miRNAs ranked via *p*-value are shown. In the case of *p*-value ties, miRNAs with the largest median difference relative to the transcriptome are shown. (**G**,**H**) As in (**E**,**F**), except analyzing the target genes of miRNAs depicted in panel (**D**).

To investigate whether miRNAs were associated with K^+^-induced post-transcriptional dynamics, small RNA sequencing was next conducted to identify depolarization-sensitive miRNAs for further analysis. Differential expression analysis identified 257 (128 upregulated, 129 downregulated) significantly altered mature miRNAs immediately after depolarization, whereas 108 (73 upregulated, 35 downregulated) were identified 2 h post-stimulus (Figure 6A,B). In contrast to mRNA and RPF changes, differential expression of miRNAs showed a higher degree of overlap between experimental time-points, with 86 (30.8%) mature miRNAs persistently modulated (Figure S10).

We next examined whether mRNAs targeted by depolarisation-sensitive miRNAs were subjected to disproportionate post-transcriptional responses. To focus this analysis on brain-expressed miRNAs, we intersected significantly differentially expressed miRNAs with the top quintile of brain-expressed miRNAs reported by the miRMine database [31]. A refined list of 54 (39 upregulated, 15 downregulated) brain-enriched, K^+^-responsive miRNAs were subsequently identified immediately after depolarisation for further analysis (Figure 6C,D). We firstly examined the aggregate effect of the 39 upregulated miRNAs on target gene expression, stratified by the number of expressed miRNA recognition elements predicted by the TargetScan database [32]. Strong repression of target gene abundance was subsequently observed relative to the entire transcriptome, in which the magnitude of repression was augmented by the number of predicted binding sites for all genes with > 1 site (*p* < 5.118e^−14^; Figure 6E). In contrast, no substantial biases in target gene RPF expression were detected (*p* ≥ 0.011), resulting in a cumulative increase in TE for all genes with > 1 site (*p* ≤ 5.919e^−7^; Figure 6E). When targets of these miRNAs were examined individually, loss of mRNA abundance without corresponding changes in RPF expression was again observed, with particularly strong repression observed for targets of miR-181b/c/d-5p, miR-221/222-3p, miR-454-3p, miR-30a/e-5p and miR-26a/b-5p (p_mRNA_ < 2.2e^−16^, p_RPF_ ≥ 5.62e^−3^, p_TE_ ≤ 5.78e^−10^; Figure 6F). Overall, these results suggest that mRNAs targeted by miRNAs upregulated in response to K^+^ stimulation are predominantly regulated at the level of mRNA abundance. Furthermore, these effects appear to occur in a temporally restricted manner, given that similar analysis conducted 2 h post-depolarisation uncovered minimal significant results (Figure S11).

Analysis of mRNAs targeted by the 15 downregulated miRNAs produced comparatively discordant results. Specifically, changes in mRNA abundance did not show any relationship with the number of predicted miRNA binding sites, while no significant changes in RPF expression and TE were observed (*p* ≥ 0.127; Figure 6G). Interestingly, mRNAs targeted by miR-744-5p and miR-331-3p were subjected to an increase in mRNA abundance (*p* ≤ 4.09e^−9^) and decrease in TE (*p* ≤ 0.015) as anticipated (Figure 6H), however, opposite effects were observed for targets of miR-30b-5p, miR-107/103a-3p and miR-411-5p (Figure 6H). For miR-30b-5p in particular, we suspect these dynamics resulted from upregulation of miR-30a/e-5p, all of which bear the same seed region (Figure 6C).

### 2.7. Differential Expression of tRNA-Derived Small RNA Fragments After Depolarisation

**Figure 7:**
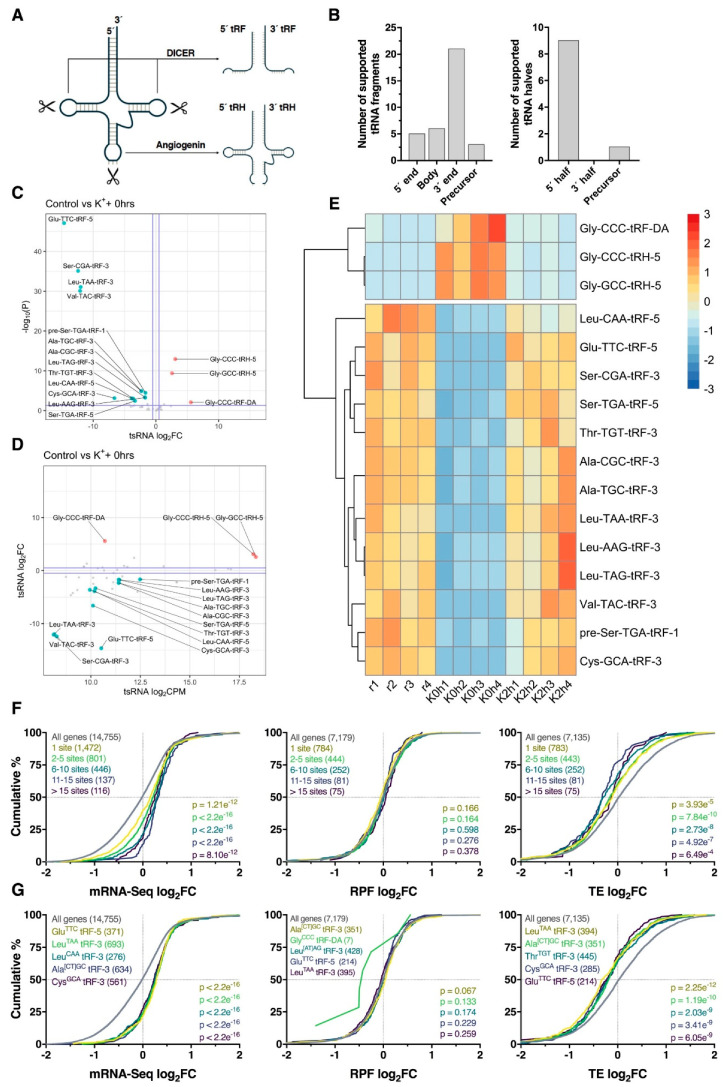
Differential processing of tRNA-derived small RNA fragments. (**A**) Schematic overview of cleavage sites required for processing of tRNAs into ~14–30 nt tRNA-derived fragments (tRFs) and ~28–36 nt tRNA-derived halves (tRHs; also known as tiRNAs). (**B**) Bar plots summarizing the locations at which tsRNAs were cleaved from mature tRNAs or precursor tRNAs. (**C**) Volcano plot comparing log_2_ fold change (log_2_FC) and –log_10_ *p*-values for tsRNAs immediately after the depolarization paradigm. Note the bias towards downregulation of tRFs. Significantly upregulated and downregulated tsRNAs are marked in red and blue, respectively, with Benjamini–Hochberg FDR < 0.05 and log_2_FC > |± 0.5| considered significant. Horizontal line represents nominal *p* < 0.05. Suffixes denote location of origin (1 = 5′ precursor sequence, 3 = 3′ mature sequence, 5 = 5′ mature sequence and DA = mature tRNA D and A loops). (**D**) Scatter plot comparing tsRNA log_2_FC and average counts per million (CPM). (**E**) Heat map depicting tsRNA CPM across all samples for significantly regulated tsRNAs identified in panel (**C**). Each cell corresponds to the CPM standard deviation relative to the row-wise mean, with red corresponding to high expression, and blue to low expression. (**F**) Cumulative distribution profiles of mRNAs collectively targeted by all significantly downregulated tRFs, after stratifying for the number of predicted binding sites. Groups were compared to the entire transcriptome via two-sided Kolmogorov–Smirnov test, with *p*-values reported (bottom right). (**G**) As in (**F**), except stratified for targets of individual tRFs rather than analyzing them in combination. Top five tRFs ranked via significance are shown. In the case of *p*-value ties, tRFs with the largest median difference relative to genes with no binding site were plotted.

Recent studies have shown that endonucleolytic cleavage of tRNA hairpin structures can produce tRNA-derived small RNAs (tsRNAs), a novel class of small RNA consisting of 14–30 nt tRNA fragments (tRFs) and 28–36 nt tRNA halves (tRHs; Figure 7A). Since these molecules exhibit potential to regulate mRNAs post-transcriptionally [33,34], we next examined small RNA-Seq data for depolarization-associated tsRNA expression using ‘tDR Mapper’. In total, 46 robustly expressed (CPM > 100) tsRNAs were identified, of which 35 were tRFs and 10 tRHs. Analysis of read coverage revealed that tRFs were predominantly processed from the 3′ end of parent tRNAs (21, 60%), whereas tRHs were generally derived from tRNA 5′ ends (9, 90%), consistent with current literature pertaining to tRNA cleavage [33,34] (Figure 7B). Differential expression analysis revealed 16 significantly regulated tsRNAs immediately after K^+^ stimulation, consisting of 14 downregulated tRFs, one upregulated tRF and two upregulated tRHs (Figure 7C–E). Interestingly, these tsRNAs all returned to baseline levels 2 h post-stimulus (Figure 7E), and furthermore, no significant differentially expressed tsRNAs were detected at this time-point, suggesting tsRNA differential processing was temporally restricted.

Since tRFs have previously shown potential to act as miRNA analogues [33,34], we next identified potential tRF–mRNA interactions involving significantly regulated tRFs for further analysis. Using ‘RNAhybrid’, we identified all potential tRF-3′UTR interactions using nucleotides 2–8 of the tRF as the seed region. Surprisingly, these tRF seed regions exhibited no overlap with the seed regions of known human miRNAs, with the exception of Ala[T/C]^GC^-tRF-3, which shares a predicted seed region (5′-CCCCGGC-3′) with the poorly conserved miR-4707-5p. Analysis of all predicted target genes revealed that regardless of the number of binding sites, loss of tRF expression corresponded to an increase in target gene abundance (*p* ≤ 8.1e^−12^) without any alteration of RPF log2FC (*p* ≥ 0.164) distribution profiles (Figure 7F). This effect was most pronounced when predicted targets of Glu^TTC^-tRF-5, Leu^TAA^-tRF-3, Leu^CAA^-tRF-3, Ala[CT]^GC^-tRF-3 and Cys^GCA^-tRF-3 were analyzed (all *p* < 2.2e^−16^; Figure 7G). Given the weak RPF level responses, decreases in TE were subsequently observed.

## 3. Discussion

Activity-dependent mRNA translation is crucial for the regulation and fine-tuning of synaptic circuitry in the brain. Previous studies have shown that overall translation is highly responsive to neuronal activity and experience-dependent plasticity [12,35,36]; however, discrete quantification of gene-level translation is necessary for a detailed mechanistic understanding of neuronal function. In the current study, we conducted transcriptome-wide translational profiling to investigate the dynamics of depolarization-associated translation and explore key sequence-dependent regulatory systems involved. Our analyses revealed that translational remodeling is extensive after repeated K^+^ stimulation, with the most prominent changes to ribosomal occupancy occurring immediately after depolarization. Functional analysis additionally identified transient enrichment of genes associated with neuron-specific functions and cellular components, after which production of genes related to nucleotide-binding and transcription was prioritized. Collectively, these findings are consistent with previously documented alterations to neuronal translation associated with K^+^ stimulation [35,37,38,39] and other excitatory stimuli [16,40]. More broadly, the functional partition between translational profiles at both experimental time-points emphasizes the importance of early translational regulation after neuronal excitation, supporting the recent identification of a critical 2-h time-window during long-term potentiation (LTP) associated with extensive translational remodeling [41].

Transcriptome-wide analysis of mRNA expression identified considerable changes to mRNA abundance, a largely anticipated finding given the previously reported transcriptomic alterations in paradigms of neuronal excitation [42,43]. Specifically, recent work has identified activity-regulated transcription factors and enhancers—such as MEF2, CBP and NPAS4—which mediate unique gene expression programs tailored to specific patterns of neuronal activity, including K^+^ depolarization [42,43,44,45]. A consistent feature of these studies is the upregulation of critical activity-associated genes with known importance in the regulation of neuronal plasticity. Interestingly, while many of these genes were upregulated at the mRNA level at one or both time-points in our study, a subset were subjected to increased translation (i.e., ERG1, FOS, IER2, amongst others), whereas others exhibited no response (i.e., BDNF, NPAS4 and ARC, amongst others). The latter group is particularly surprising, given that activity-dependent translation of some of these genes has been documented previously [15,46,47], suggesting post-transcriptional regulatory mechanisms determined translational regulation of these activity-associated genes in our study. Prospective studies examining transcriptional profiles associated with unique patterns of neuronal activity will, therefore, benefit from incorporation of ribosome profiling to map the ratio of transcriptional and translational change for activity-associated genes and explore post-transcriptional regulatory events of biological significance.

By comparing fluctuations in mRNA abundance and translation, we uncovered extensive changes to translational efficiency which were most pronounced immediately after depolarization. Further analysis revealed that a large proportion of genes at this time-point were subjected to regulation exclusively at the mRNA or RPF levels, but not both, suggesting depolarization-associated changes in mRNA abundance and translation were functionally partitioned. We suspect this buffering effect serves to maintain protein output during the course of perturbation irrespective of large changes in transcription, a phenomenon previously observed in yeast and humans [48,49,50,51]. The strengthened correlation between mRNA abundance and translation 2 h after stimulation suggests this buffering effect is transient, with more-conventional post-transcriptional dynamics progressively restored post-stimulus. These results are consistent with a recent study in which changes in mRNA abundance and translation were largely proportional after K^+^ stimulation of primary mouse cortical neurons for 3 h [38]. However, divergent patterns of mRNA abundance and translation have additionally been reported after chemical LTP in mouse hippocampal neurons [41], stressing the context-dependent nature of post-transcriptional regulatory programs associated with neuronal stimulation. While future studies with greater temporal resolution may uncover the factors involved in this transition, we suspect transient depolarization-associated post-transcriptional buffering could explain why sequence features were initially associated with mRNA abundance but became more associated with translation 2 h post-stimulus.

The detection of such a large post-transcriptional buffering effect draws attention to the regulatory events mediating the division between mRNA abundance and translation. Although we suspect the sequence-specific mechanisms investigated in this study contribute to this, additional post-transcriptional factors likely play a more critical role. One distinct possibility is that K^+^ stimulation results in extensive reorganization of mRNA spatial distribution profiles, in which trafficking of mRNAs between storage granules (such as *p*-bodies and stress granules) and translationally active compartments could offer a mechanism through which mRNA abundance and translational capacity could be independently regulated [52]. P-bodies in particular have been observed to undergo dynamic remodeling at the post-synapse [53], and recently, bidirectional movement of mRNAs between these granules and polysomes has been described after neuronal excitation [54]. Such a mechanism would be consistent with the large proportion of genes subjected to changes in ribosome occupancy, with little detectable change in abundance. P-bodies also frequently interact with neuronal granules associated with mRNA trafficking [53], indicating that these structures may also offer a site in which newly synthesized mRNAs may be stored for delayed translation, potentially contributing to the buffering effect observed at this time-point. While future studies that allow for the spatial distribution mRNA profiles associated with excitatory stimuli could further refine this model, we suspect a significant proportion of these dynamics are attributable to the action of RNA-binding proteins, particularly translational enhancers and repressors, and therefore, quantification of RPB–mRNA interactions would also add to our understanding of the complexes involved.

Many miRNAs are known to regulate structural and physiological features of neurons associated with excitability [30]; however, their behavior at the post-transcriptional level during neuronal excitation remains obscure. Canonically, miRNAs are thought to reduce translational competency and abundance of target mRNAs by triggering sequential deadenylation, decapping and exonucleolytic degradation [30]; however, a subset of miRNA–mRNA interactions resulting in translational regulation without mRNA degradation have recently been uncovered in the neural setting [55,56,57,58]. Our findings support the canonical mechanism of miRNA-mediated mRNA repression, as upregulation of miRNAs was generally associated with downregulation of target mRNA abundance in proportion to the number of miRNA recognition elements. Interestingly, we failed to observe initial miRNA-associated changes in RPF expression that have previously been reported after exogenous miRNA modulation [59,60]; however, we suspect this was due to the pervasive post-transcriptional buffering detected at this time-point. This further raises the possibility that translationally active mRNAs were resistant to miRNA-associated changes in mRNA abundance, in which case we suspect additional post-transcriptional regulatory factors contribute, such as the spatial organization of mRNAs [52], m6A methylation [61], alternative polyadenylation [62] or competitive binding by RNA binding proteins [63,64,65]. Nevertheless, these results are particularly interesting and support further investigation into the interplay between excitation-induced miRNAs and translationally active mRNAs.

Another interesting feature of K^+^-associated miRNA dynamics was the prompt and extensive remodeling of miRNA expression at both experimental time-points, indicating that miRNAs themselves are subjected to fine-tuning after repeated stimulation. Although rapid, excitation-associated miRNA biogenesis can be explained by known mechanisms, including activity-dependent transcription of miRNA genes [66,67] and localized processing of mature miRNAs [28,29], swift downregulation of mature miRNAs was not expected given the known stability of miRISC complexes [68]. One mechanism capable of circumventing this is expulsion via exosome release, a phenomenon previously associated with downregulation of neurite-enriched miRNAs after K^+^-stimulation of SH-SY5Y cells [69]. In support of this hypothesis, we detected significant downregulation of miR-1228-3p and miR-320a, two miRNAs detected within excitation-associated exosomes in the aforementioned study. Another mechanism of interest is target-directed miRNA degradation (TDMD), in which extensive complementarity between a miRNA and its target leads to miRNA decay via 3′ trimming or the untemplated addition of A residues [70,71,72]. Interestingly, TDMD has recently been demonstrated in neuronal models, in which potent degradation of important miRNAs, such as miR-124, miR-128, miR-132 and miR-138, has been detected [70,71,72], of which miR-132 and miR-138 were downregulated immediately after stimulation in our study. However, the temporal dynamics of this mechanism are yet to be elucidated, particularly in the context of neuronal excitation.

Analysis of small RNA data also uncovered the expression of tsRNAs, which exhibited temporally restricted differential expression immediately after repeated K^+^ stimulation. Furthermore, significant downregulation of tRFs was associated with increased expression of mRNAs with predicted 3′UTR binding sites, indicating a potential role in the post-transcriptional regulation of mRNA. Although tsRNAs were originally characterized as byproducts of tRNA degradation [73], mounting evidence suggests that tRNA fragments engage in post-transcriptional repression of complementary mRNAs, acting as miRNA analogues [33,34]. Our results in particular support an emerging body of studies in which tRFs have been shown to associate with AGO paralogs, bind mRNA 3′UTR sequences via a 5′ miRNA-like seed region and repress mRNA expression [74,75,76,77]. Interestingly, such as role has been described for Leu^TAA^-tRF-3 [76], which was significantly downregulated and associated with increased mRNA abundance in our study. However, additional studies suggest that tRFs can also regulate repression of mRNAs through atypical binding patterns, indicating that their post-transcriptional function is complex and requires further investigation [78,79]. Nonetheless, the identification of tRFs in our study remains a particularly interesting finding and represents some of the first evidence of tRF differential expression in stimulated neuronal cells, warranting additional study into the dynamics of these molecules after neuronal depolarization. Although the differential expression of tRHs was another interesting aspect of these data, these molecules are thought to displace translational machinery by binding mRNA g-quadruplex structures [33,34], hence limiting the investigation of potential sequence dependent regulation in our data.

In summary, our findings suggest that post-transcriptional dynamics are substantially altered following repeated K^+^ stimulation, in which sequence-dependent regulatory elements are considerably implicated. Further dissection of the spatial factors involved in this system will likely prove critical in elucidating the true manner in which these processes are coordinated. Likewise, integrated analysis of mRNA 3′UTR expression, m6A modifications and changes in protein functional status, amongst other systems, is required to further decipher the intricacies of the neuronal post-transcriptional environment, which will substantially improve our understanding of the molecular events underpinning learning, memory, cognition and psychiatric disorders.

## 4. Materials and Methods

### 4.1. Cell Culture

Human SH-SY5Y neuroblastoma cells were grown and sustained in Dulbecco’s Modified Eagle’s Medium (DMEM, HyClone, Logan UT, USA) supplemented with 10% heat-inactivated fetal bovine serum (Bovogen Biologicals, Essendon, VIC, Australia), 2% HEPES (Gibco, Carlsbad, CA, USA) and 1% L-glutamine (Invitrogen, Carlsbad, CA, USA). Cultures were maintained at 37 °C in a 95% oxygen, 5% carbon dioxide, 90% humidity atmosphere and passaged regularly via washing with phosphate buffered saline (PBS; Gibco) and trypsinization (0.1% trypsin-EDTA in PBS; Gibco). All cells used in this study were passage 7 at the time of harvest.

### 4.2. Neuronal Differentiation

SH-SY5Y cultures were neuronally differentiated using all-trans retinoic acid (ATRA, Sigma-Aldrich, St. Louis, MO, USA) [80]. One day prior to differentiation (day -1), cells were seeded at a density of 25,000 cells/cm^2^ in T175 culture flasks. The next day (day 0), standard culture medium was replaced with ATRA-supplemented medium (10µM final) and cells were wrapped in foil to protect them from light exposure. The ATRA-medium was subsequently replaced on days 2, 4 and 6, after which the methods were continued as described on day 7. Successful differentiation was confirmed by examining neurite outgrowth and establishing the upregulation of known neuronal marker genes relative to naïve cells via mRNA sequencing (See Figure 1B–D).

### 4.3. Membrane Depolarisation

Whole cell depolarization was induced by incubating cells in Hank’s Balanced Salt Solution (HBSS; 35 mM NaCl, 0.6 mM MgSO_4_.7H_2_O, 2.5 mM CaCl_2_.2H_2_O, 10 mM HEPES, 6 mM Glucose) supplemented with depolarizing concentrations of KCl (100 mM). Depolarization regimens consisted of 3 min exposure to 37 °C HBSS, followed by 10 min recovery at standard culturing conditions. A total of 4 consecutive stimulus and recovery cycles were employed to simulate patterns of repetitive excitation thought to be important for induction of neuronal long-term potentiation. Cells were consequently harvested either immediately or 2 h after the final stimulus and recovery cycle to resolve early and late expression changes. All treatments were performed in biological quadruplicate.

### 4.4. Ribosome Profiling

Ribosome profiling was conducted via the Illumina TruSeq Ribo Profile kit (H/M/R), with minor amendments to the manufacturer’s protocol. To suspend translational activity, cells were incubated with a warm culture medium supplemented with 0.1mg/mL cycloheximide (CHX) for 1 min. After aspirating the CHX medium and washing cells with ice-cold CHX-supplemented PBS, 1 mL mammalian lysis buffer (containing 200 µL 5× mammalian polysome buffer, 2 µL CHX and 10 units (U) DNase I) was added to each culture and cells were extensively scraped on ice. Ribosome foot-printing was performed by adding 90U RNase I to 300 µL clarified lysate and incubating samples at room temperature for 45 min, after which reactions were stopped with 15 µL SUPERase inhibitor (Invitrogen). Ribosome-protected RNA fragments (RPFs) were then enriched using MicroSpin S-400 columns (GE Life Sciences, Marlborough, MA, USA), prior to ribosomal RNA depletion, RPF size selection, end repair, adapter ligation, reverse transcription and PAGE purification, conducted according to the manufacturer’s instructions. RPF cDNA libraries were subsequently produced with 12× PCR cycles, following which adapter dimers were removed via 8% native polyacrylamide gel prior to library normalization and pooling. RPF libraries were then sequenced using the Illumina NextSeq 500 system, with a total of 76 single-end cycles performed. See Supplementary Methods for further details.

### 4.5. mRNA and Small RNA Sequencing

Total RNA was extracted from 300 µL clarified lysate produced from the ribosome profiling protocol using 1mL TRIzol reagent (ThermoFisher, Waltham, MA, USA) as per the manufacturer’s instructions, with RNA precipitated at −30 °C for 2 h to enhance yield. For each sample, 1 µg of high quality (RIN ≥ 8.5) total RNA was subjected to either poly(A)-enriched mRNA sequencing using the Illumina TruSeq Stranded mRNA Library Preparation Kit, or small RNA sequencing using the Illumina TruSeq Small RNA Library Preparation Kit (both according to the manufacturer’s instructions). These libraries were subjected to 151 (mRNA) or 76 (small RNA) single-end sequencing cycles using the Illumina NextSeq 500. See Supplementary Methods for further details.

### 4.6. Processing of Sequencing Data and Differential Expression Analysis

Raw BCL files were demultiplexed and converted to fastq format using ‘Bcl2fastq’ (v2.2; Illumina), after which data quality was assessed using ‘FastQC’ (v0.11.5). Sequencing adapters and low quality 3′ nucleotides (Phred33 score < 28) were then trimmed using ‘Cutadapt’ (v1.14) [81]. For Ribo-Seq libraries, single 5′ nucleotides were additionally trimmed (these are often untemplated additions during library preparation [82]) and abundant short non-coding RNAs (rRNA, snRNA, snoRNA and miRNA) were identified and discarded using ‘Bowtie2′ (v2.2.6) [83]. All libraries were then mapped to the reference genome (hg38 assembly) using ‘TopHat2′ (v2.1.1; Ribo-Seq and mRNA-Seq) [84] or ‘Bowtie2′ (small RNA), prior to read-counting via ‘HTSeq’ (v0.7.2) [85]. Feature mapping rates were analyzed using ‘RSeQC’ (v2.6.4) [86], while metagene analysis and subcodon phasing were preformed via the ‘metagene’ and ‘phase_by_size’ scripts of the ‘Plastid’ python library [87], respectively.

For each library preparation method, count normalization and differential expression were performed using ‘EdgeR’ (v3.8) [88]. Raw count data from each sample was firstly merged into a single matrix and normalized to sequencing depth (counts-per-million, CPM), after which genes with consistently low counts were removed by employing a CPM threshold. Normalization factors were then calculated via TMM method, dispersion was estimated and pairwise differential expression relative to untreated control cells was performed via exact test. Variation amongst biological replicate samples was visually assessed pre- and post-normalization via multidimensional scaling and biological coefficient of variation plots (Figures S1–S3). Changes in translational efficiency (TE) were additionally calculated using the ‘RiboDiff’ (v0.2.1) [89] package, using raw mRNA and RPF counts as inputs. For all differential expression analyses, a Benjamini–Hochberg false discovery rate <0.05 and absolute log_2_ fold change >0.5 were considered significant. See Supplementary Methods for further details and Tables S2 and S3 for all differential expression results. Raw sequencing and processed read-count data are available at the Gene Expression Omnibus (Accession: GSE155727).

### 4.7. Gene Ontology Enrichment Analysis

Analysis of enriched gene ontology terms was conducted using the ‘Toppgene’ functional enrichment web suite [90], with a Benjamini–Hochberg false discovery rate < 0.05 considered significant. 

### 4.8. Analysis of mRNA Sequence Features

For all genes with mRNA-Seq, Ribo-Seq and TE data, the most highly expressed transcript was identified from Ribo-Seq data using ‘Stringtie’ (v1.3.6) [91], then fasta files containing 5′UTR, CDS and 3′UTR sequence data for these transcripts were obtained from Ensembl Biomart (release 96) [92]. Custom bash scripts were then used to determine sequence length, GC%, rare codon frequency and the abundance of upstream start (AUG) codons. Minimum free energy (MFE) was calculated for all sequences using ‘RNAfold’ (v2.4.12) [93] prior to normalization relative to sequence length. G-quadruplex abundance was analyzed using ‘G4RNA screener’ (v0.3) [94], with a G4 neural network (G4NN) score of 0.5 used to identify significant structures. After stratification via these metrics, mRNA, RPF and TE log_2_ fold change distributions were compared via Kruskal–Wallis test, with significance accepted at *p* < 0.05.

### 4.9. Analysis of mRNA–miRNA Interactions

Significantly differentially expressed miRNAs were intersected with the top quintile of brain-expressed miRNAs assembled from the ‘miRMine’ [31] database of human miRNA expression profiles. Potentially misannotated miRNAs were then removed from further analyses using miRNA annotation data provided by the ‘TargetScan’ (v7.2) [32] database. For each remaining miRNA, computationally predicted mRNA interactions with cumulative weighted context score < –0.2 were then intersected with mRNA, RPF and TE data, and target gene expression distributions were compared to the entire transcriptome via two-sided Kolmogorov–Smirnov test, with significance accepted at *p* < 0.05. 

### 4.10. Identification and Analysis of tRNA-Derived Small RNAs

Using ‘tDR Mapper’ [95], small RNA fastq files were aligned and counted against a reference file containing mature and precursor tRNA sequences obtained from gtRNAdb (v2.0) [96]. Sequences were firstly aligned allowing for only exact matches, after which one, two and three mismatches/deletions were accepted. Read-counts for each sample were then assembled, with tsRNAs of the same species derived from parent tRNAs bearing the same anticodon and amino acid were merged to limit inclusion of identical sequences. To interrogate the capacity for tRNA-derived fragments (tRFs) to regulate mRNAs in a similar manner to miRNAs, predicted tRF-mRNA 3′UTR interactions were calculated using ‘RNAhybrid’ (v2.1.2) [97], with nucleotides 2–8 of the tRF specified as the “seed region”. Interactions with *p*-value < 0.05 were retained for further analysis. Cumulative distributions of target mRNA expression were then compared to the transcriptome as per the miRNA analysis described above. 

## Supplementary Materials

The following are available online at https://www.mdpi.com/1422-0067/21/19/7086/s1. Supplementary Methods. Figure S1: Multidimensional scaling (MDS) plots. Figure S2: Biological coefficient of variation (BCV) plots. Figure S3: MDS and BCV plots for tRNA-derived small RNAs. Figure S4: Expression dynamics of genes associated with transcription and translation. Figure S5: Relationship between mRNA dynamics and sequence features immediately after depolarization (full version). Figure S6: Effect of 5′UTR sequence features on mRNA dynamics. Figure S7: Relationship between mRNA dynamics and sequence features 2 h after depolarization (full version). Figure S8: Effect of G-quadruplex structures, upstream AUG codons and rare codon frequency on mRNA dynamics immediately after depolarization. Figure S9: Effect of G-quadruplex structures, upstream AUG codons and rare codon frequency on mRNA dynamics 2 h after depolarization. Figure S10: Venn diagram comparing expression of mature miRNAs immediately and 2 h post-stimulation. Figure S11: MiRNA–mRNA dynamics 2 h post-depolarization. Table S1: Sequencing library sizes during data processing. Table S2: Differential expression results immediately after repeated K^+^ stimulation. Table S3: Differential expression results 2 h after repeated K^+^ stimulation. Table S4: GO term analysis of genes upregulated at the RPF level immediately after repeated stimulation. Table S5: GO term analysis of genes upregulated at the RPF level 2 h after repeated stimulation. Table S6: GO term analysis of genes subjected to post-transcriptional buffering or pure translational regulation immediately after repeated stimulation.
