# Supplementary material for: Transcriptome-Wide Analysis of Interplay between mRNA Stability, Translation and Small RNAs in Response to Neuronal Membrane Depolarization
Source: Int J Mol Sci. 2020 Sep 25;21(19):7086. doi: 10.3390/ijms21197086 (PMC7582590; doi:10.3390/ijms21197086)
Supplement: Supplementary file 1 [file ijms-21-07086-s001.zip › Supplementary/sup.figs.dkiltschewskij.mcairns.revised.docx]

**Transcriptome-wide analysis of interplay between mRNA stability, translation and small RNAs**

in response to neuronal membrane depolarisation

Supplementary Figures

**
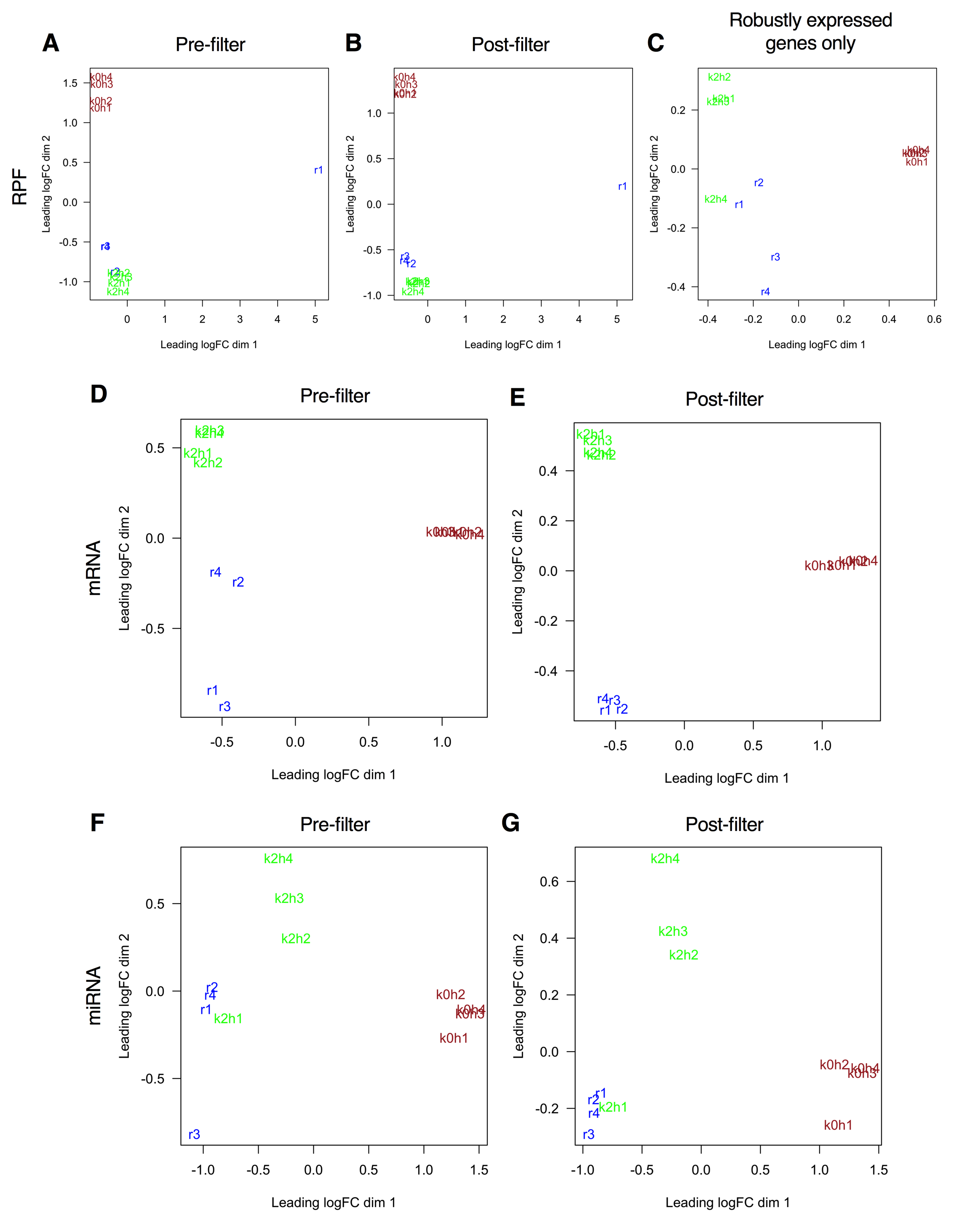
**

**Supplementary Figure 1. Multidimensional scaling plots.** Multidimensional scaling (MDS) plots for ribosome profiling **(A-C)**, mRNA **(D, E)** and small RNA **(F, G)** sequencing data both before **(A, D, F)** and after **(B, C, E, G)** removal of low read-count genes. Control samples are plotted in blue, samples harvested immediately after K^+^ treatment are presented in red, samples harvested 2 hours after K^+^ treatment are presented in green. For ribosome profiling data, control replicate 1 (r1) showed large deviation from the remaining samples before **(A)** and after **(B)** filtration due to low sequencing depth. Multidimensional scaling after removal of genes with < 100 raw counts in this sample **(C)** produced tighter clustering of all control samples, indicating this sample varied predominantly due to read depth rather than biological factors. We consequently retained this sample for downstream analysis.


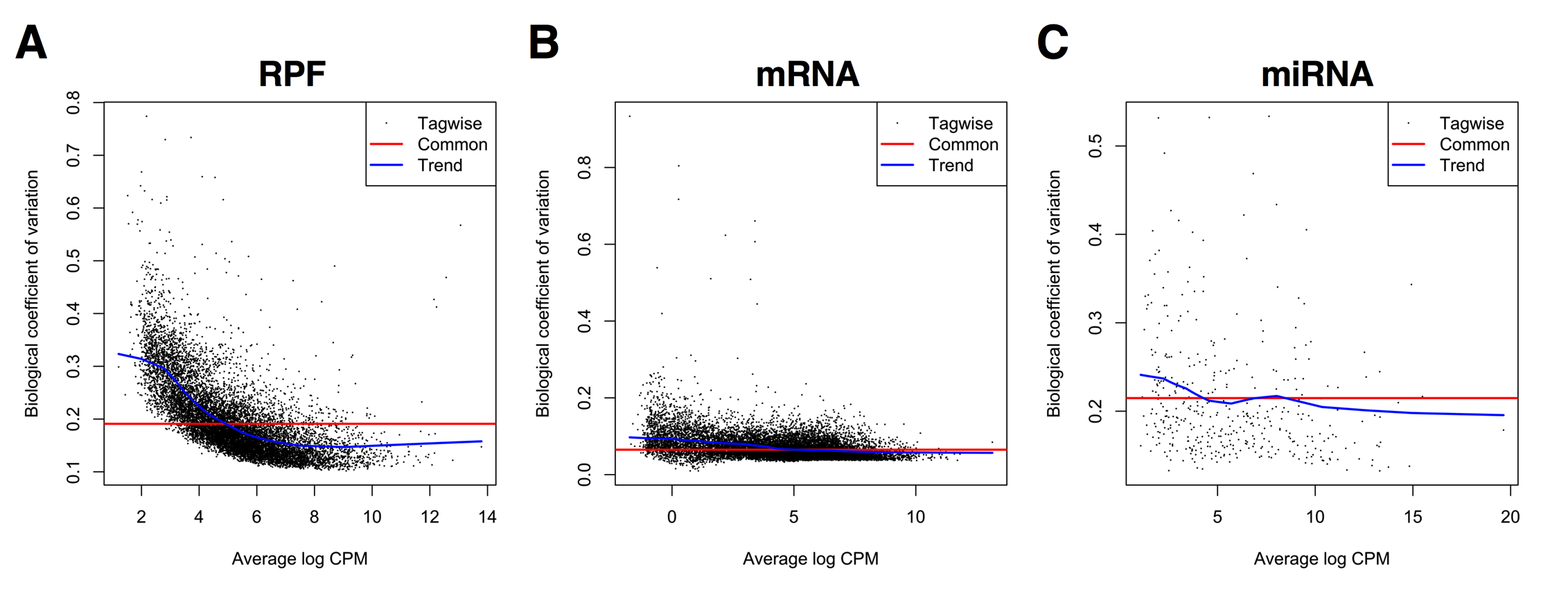


**Supplementary Figure 2. Biological coefficient of variation plots.** Biological coefficient of variation (BCV) versus average log_2_ counts per million for all genes **(A, B)** and miRNAs **(C)**. Overall, genes and miRNAs with low expression generally exhibited higher biological variation compared to their highly expressed counterparts, as indicated by the blue trendline. In addition, ribosome protected fragments exhibited the highest rates of variation compared to poly(A)-selected mRNAs and miRNAs.


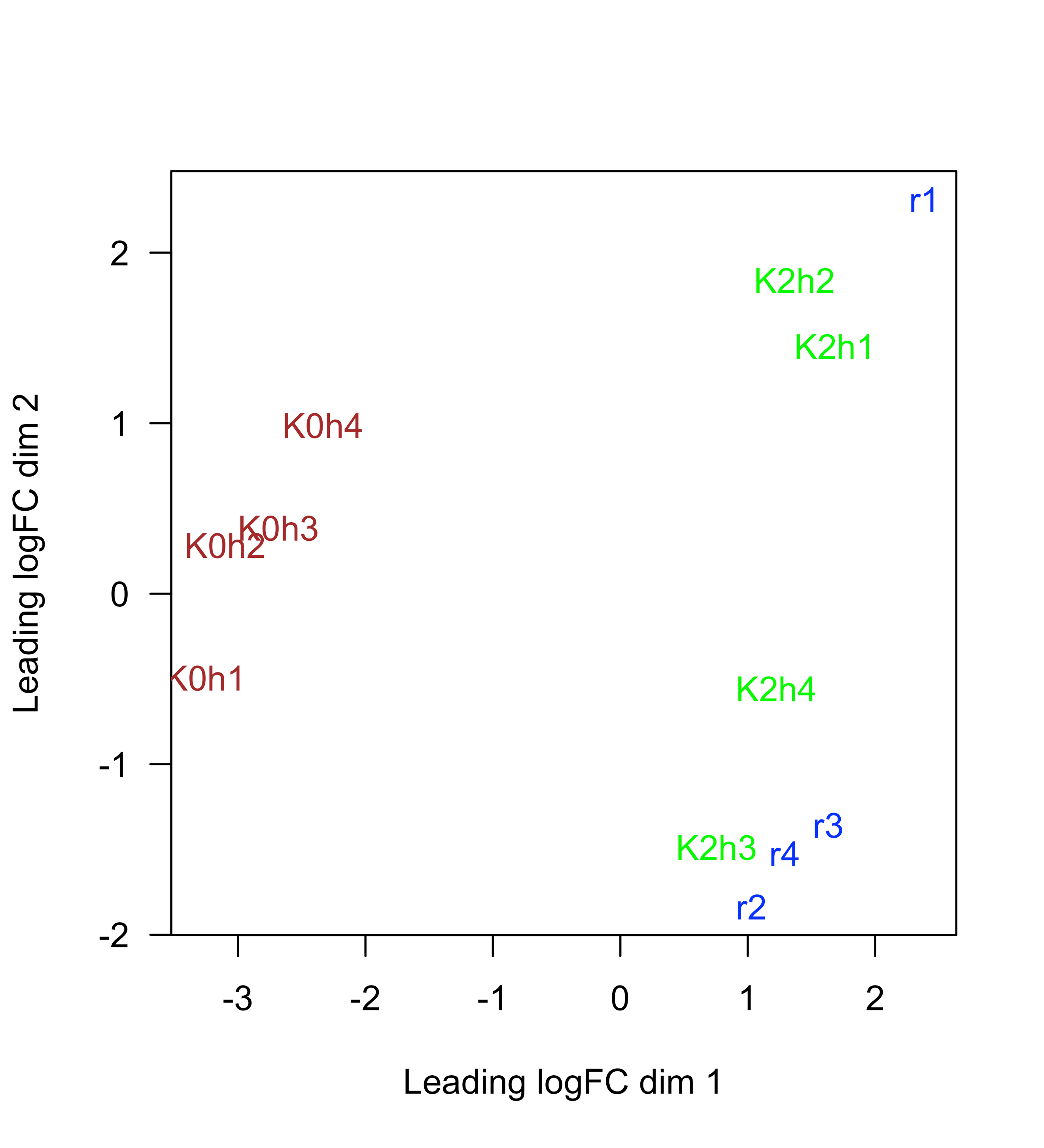

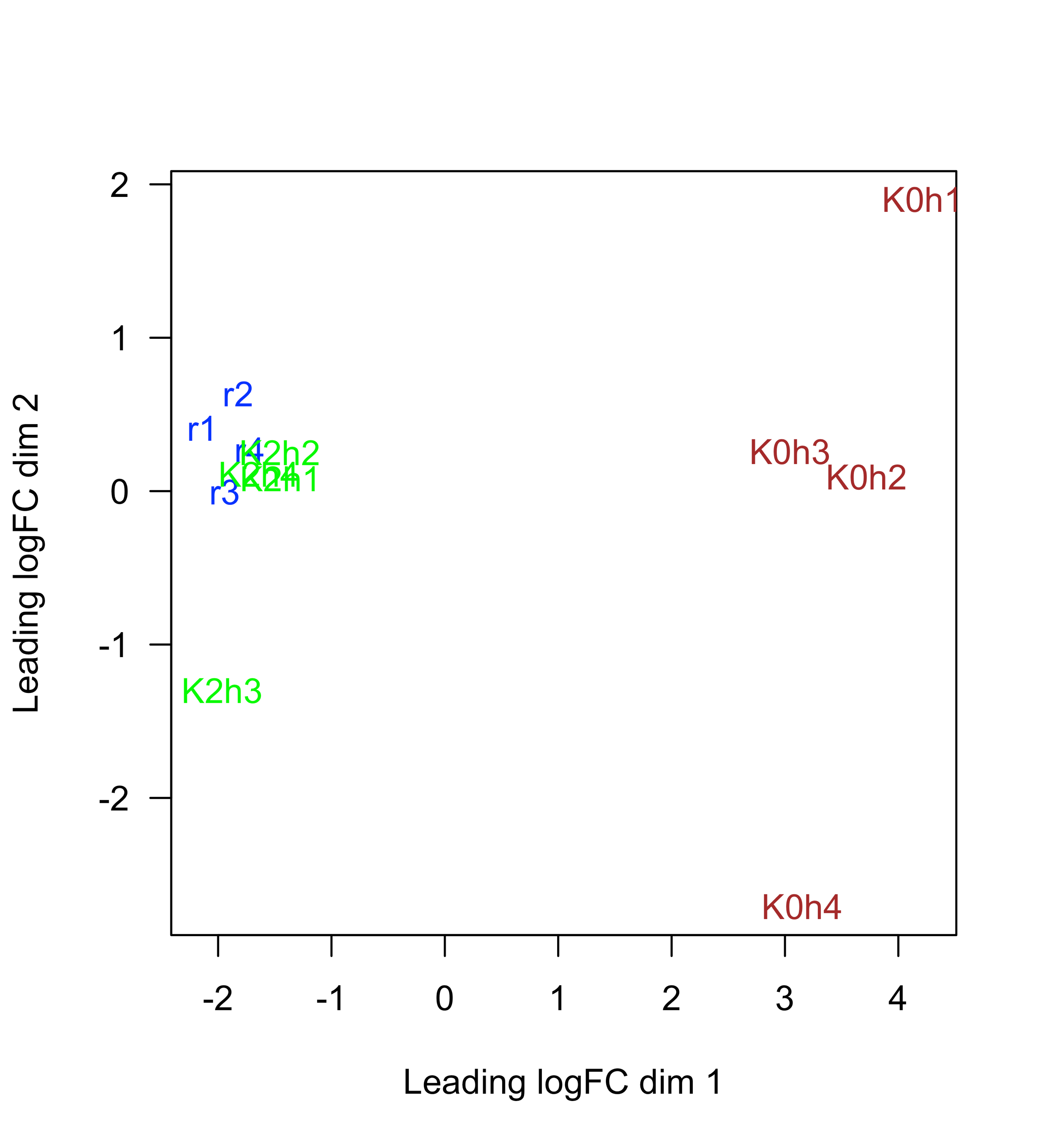

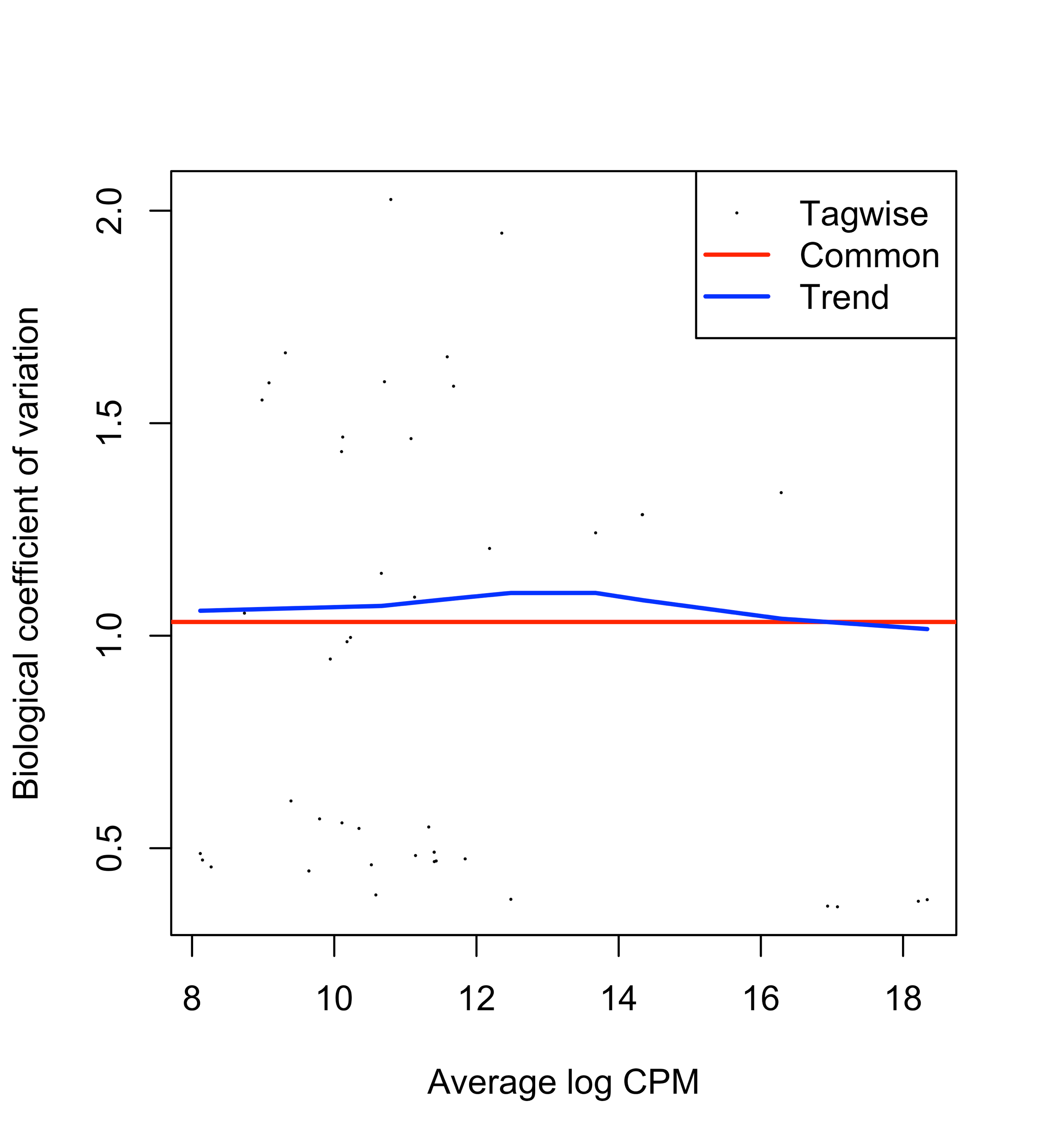


**Supplementary Figure 3. (A & B) MDS and BCV plots for tRNA-derived small RNAs.** Multidimensional scaling plots for tRNA-derived small RNAs both before **(A)** and after **(B)** removal of tsRNAs with ≤ 100 counts per million across at least 8 samples. Note the overlap of control samples and samples harvested 2 hours after K^+^ stimulation. **(C)** Biological coefficient of variation plot after removal of low read-count tsRNAs, estimation of dispersion and calculation of normalisation factors.


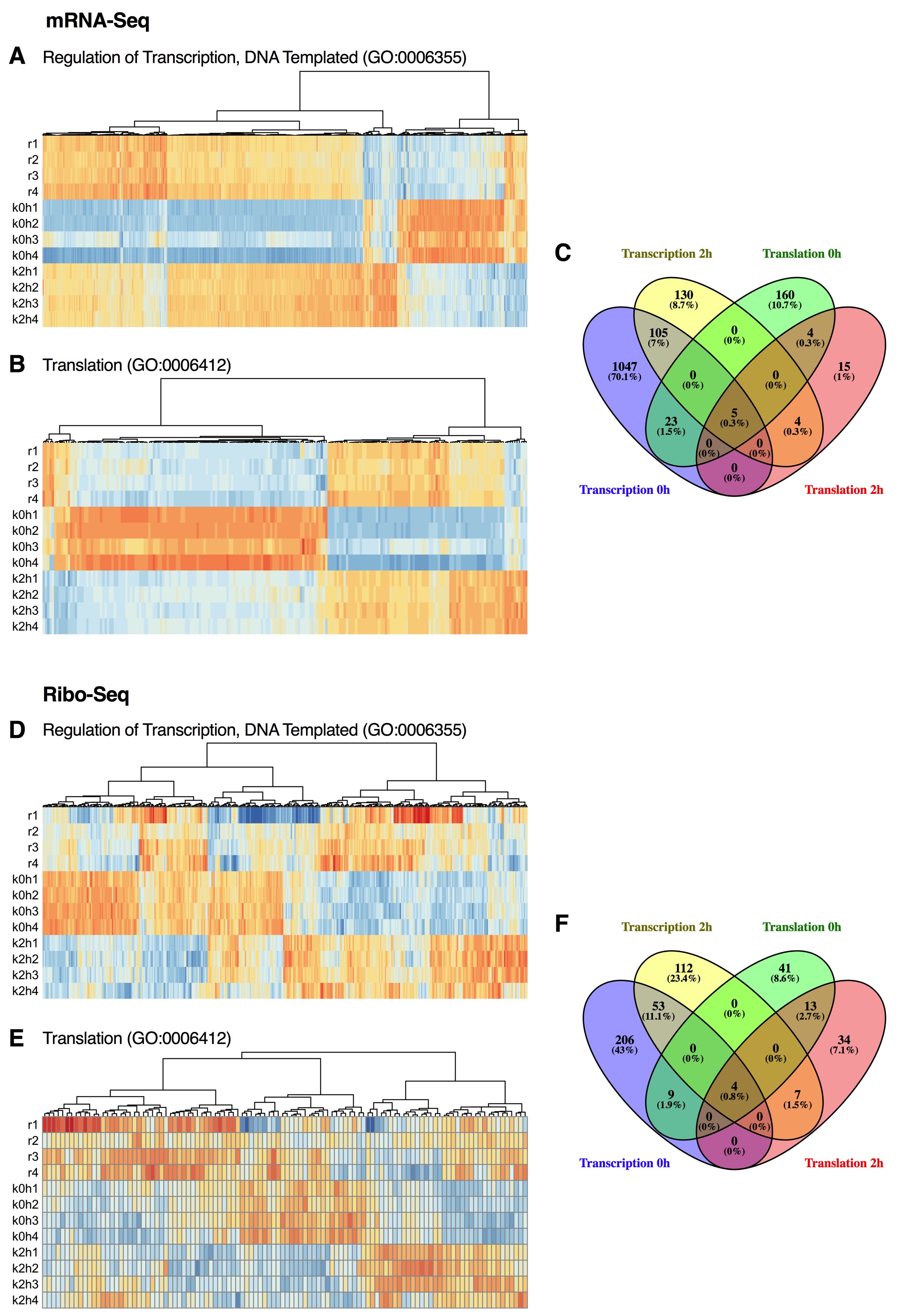


**Supplementary Figure 4. Expression dynamics of genes associated with transcription and translation. (A & B)** Heat map depicting mRNA-level expression of genes annotated to *Regulation of Transcription, DNA Templated (GO:0006355)* and *Translation (GO:0006412)* gene ontologies. All plotted genes were significantly differentially expressed at a minimum of 1 post-depolarisation time-point. Each cell corresponds to the RPKM standard deviation relative to the row-wise mean, with red corresponding to high expression, and blue low expression. **(C)** Venn diagram showing overlap of differentially expressed genes plotted in panels **(A)** and **(B)**. **(D–F)** As in **(A–C)**, except analyzing RPF-level expression.


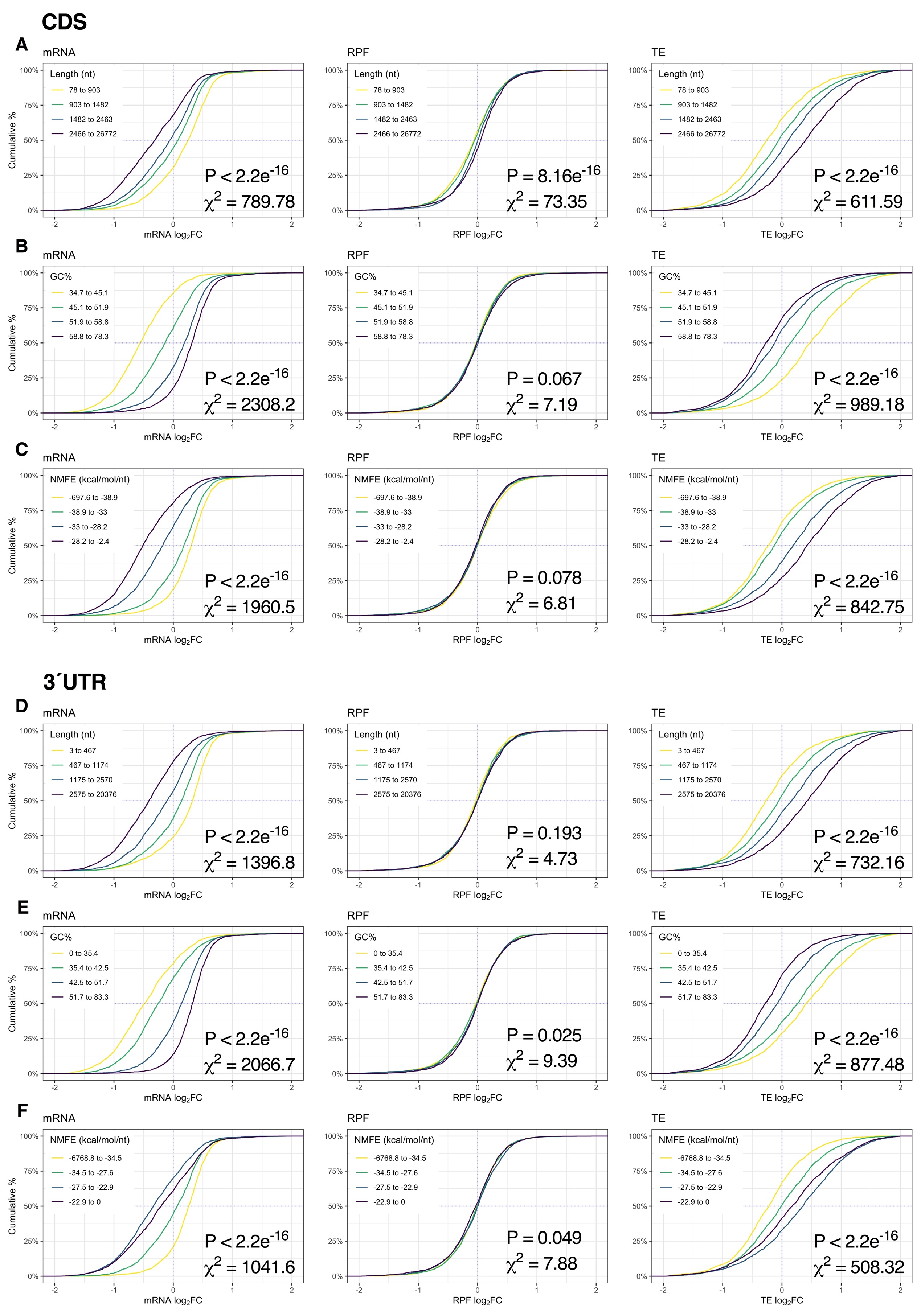
**Supplementary Figure 5. Relationship between mRNA dynamics and sequence features immediately after depolarization (full version). (A–C)** Cumulative density plots depicting changes in mRNA, RPF and TE log_2_FC after binning genes into quartiles by coding sequence length **(A)**, GC% **(B)** or minimum free energy of secondary structures normalized to sequence length (NMFE) **(C)**. All groups were compared via Kruskal-Wallis test, with associated p-values and chi-squared (𝜒^2^) test statistics reported bottom right. **(D–F)** As in **(A–C)**, except examining the same features in the 3´UTR.


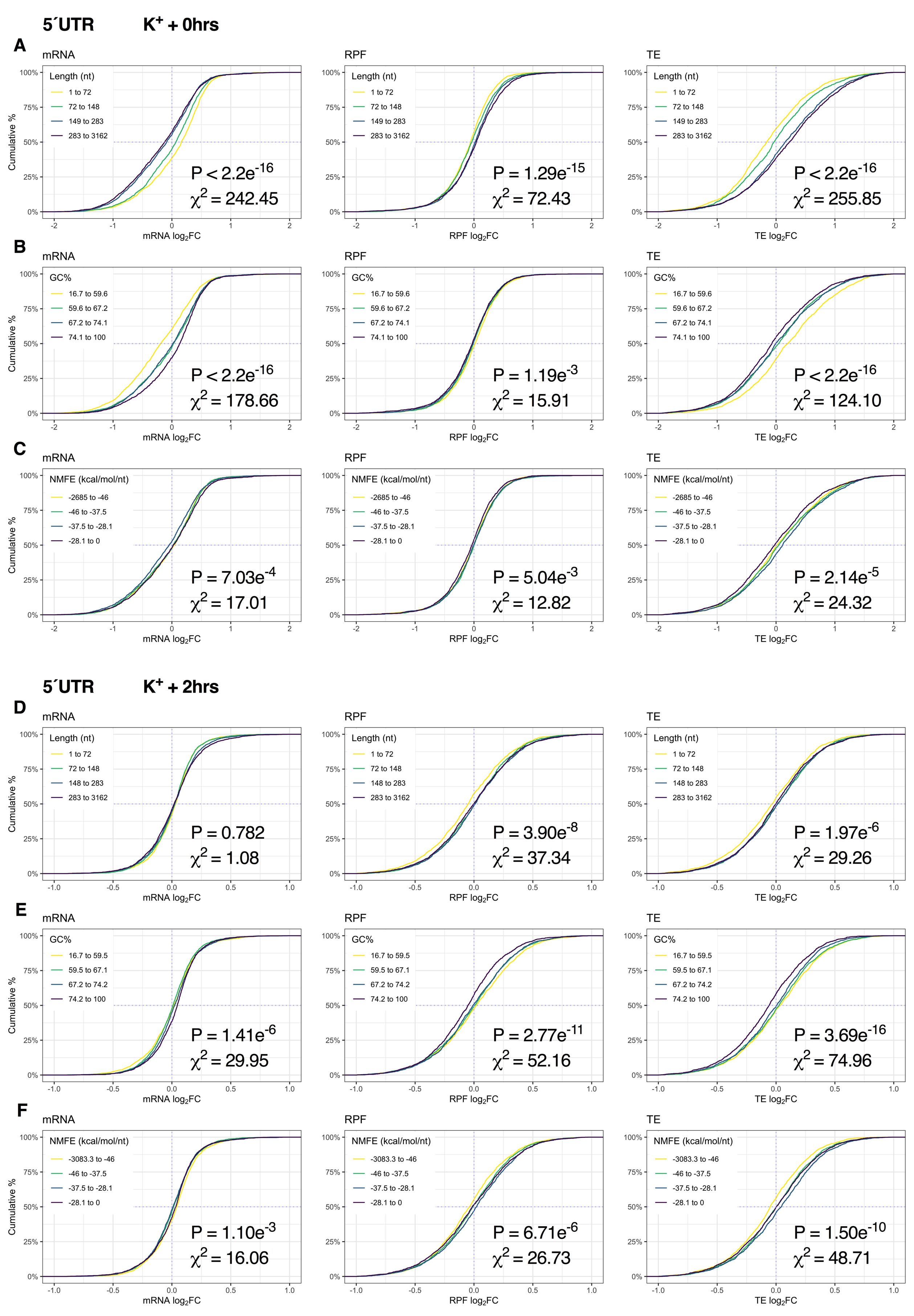


**Supplementary Figure 6. Effect of 5´UTR sequence features on mRNA dynamics.** Cumulative density plots depicting changes in mRNA, RPF and TE log_2_FC immediately **(A–C)** or 2 hours post-stimulation **(D–F)** after stratification of genes into quartiles via 5´UTR sequence length **(A & D)**, 5´UTR GC% **(B–E)** or 5´UTR minimum free energy of secondary structures normalised to sequence length (NMFE) **(C & F)**. Groups were compared via Kruskal-Wallis test, with p-values and 𝜒^2^ test statistics reported bottom right.


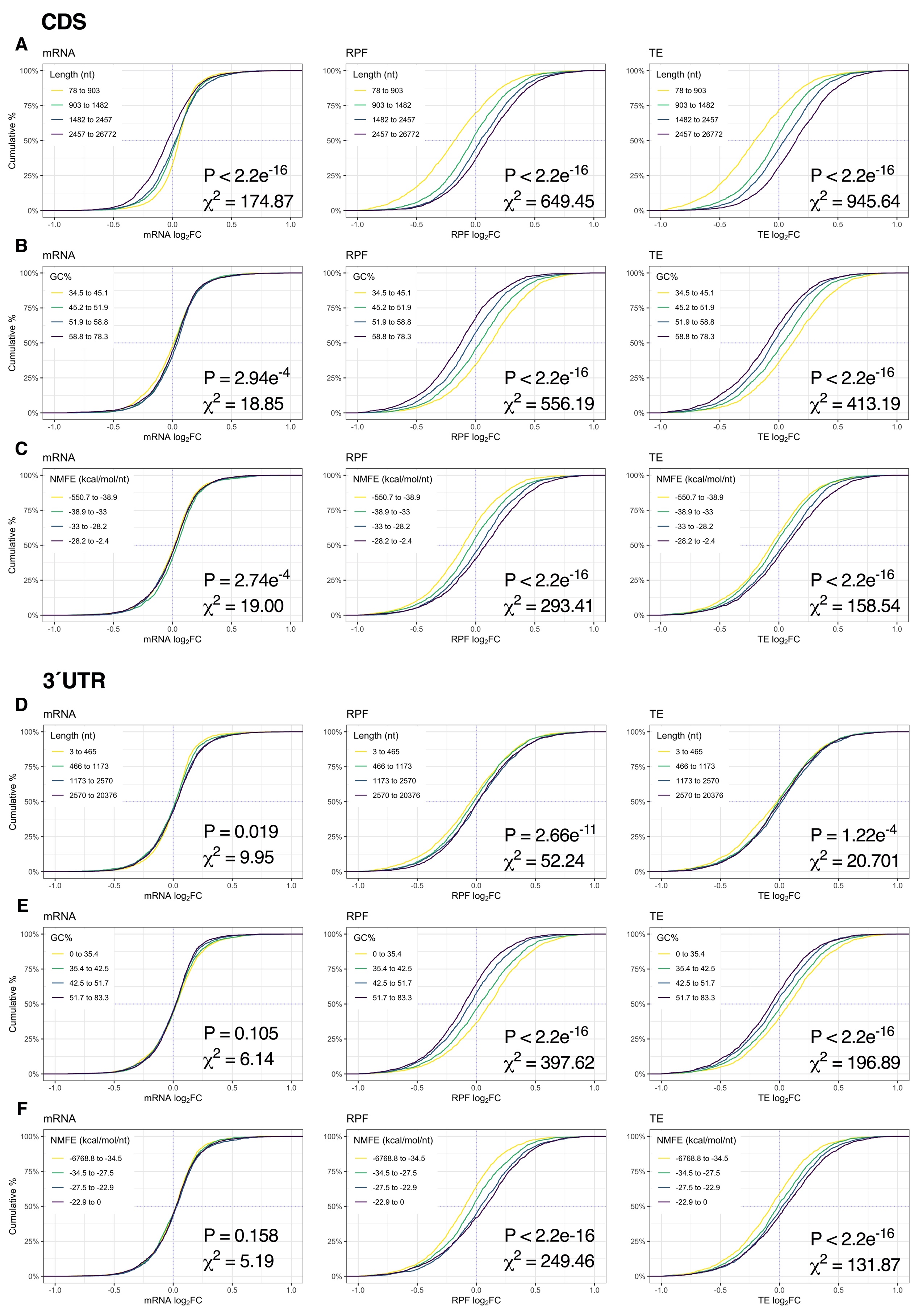
**Supplementary Figure 7. Relationship between mRNA dynamics and sequence features 2 hours after depolarization (full version). (A–C)** Cumulative density plots depicting changes in mRNA, RPF and TE log_2_FC after binning genes into quartiles by coding sequence length **(A)**, GC% **(B)** or minimum free energy of secondary structures normalized to sequence length (NMFE) **(C)**. All groups were compared via Kruskal-Wallis test, with associated p-values and chi-squared (𝜒^2^) test statistics reported bottom right. **(D–F)** As in **(A–C)**, except examining the same features in the 3´UTR.

**
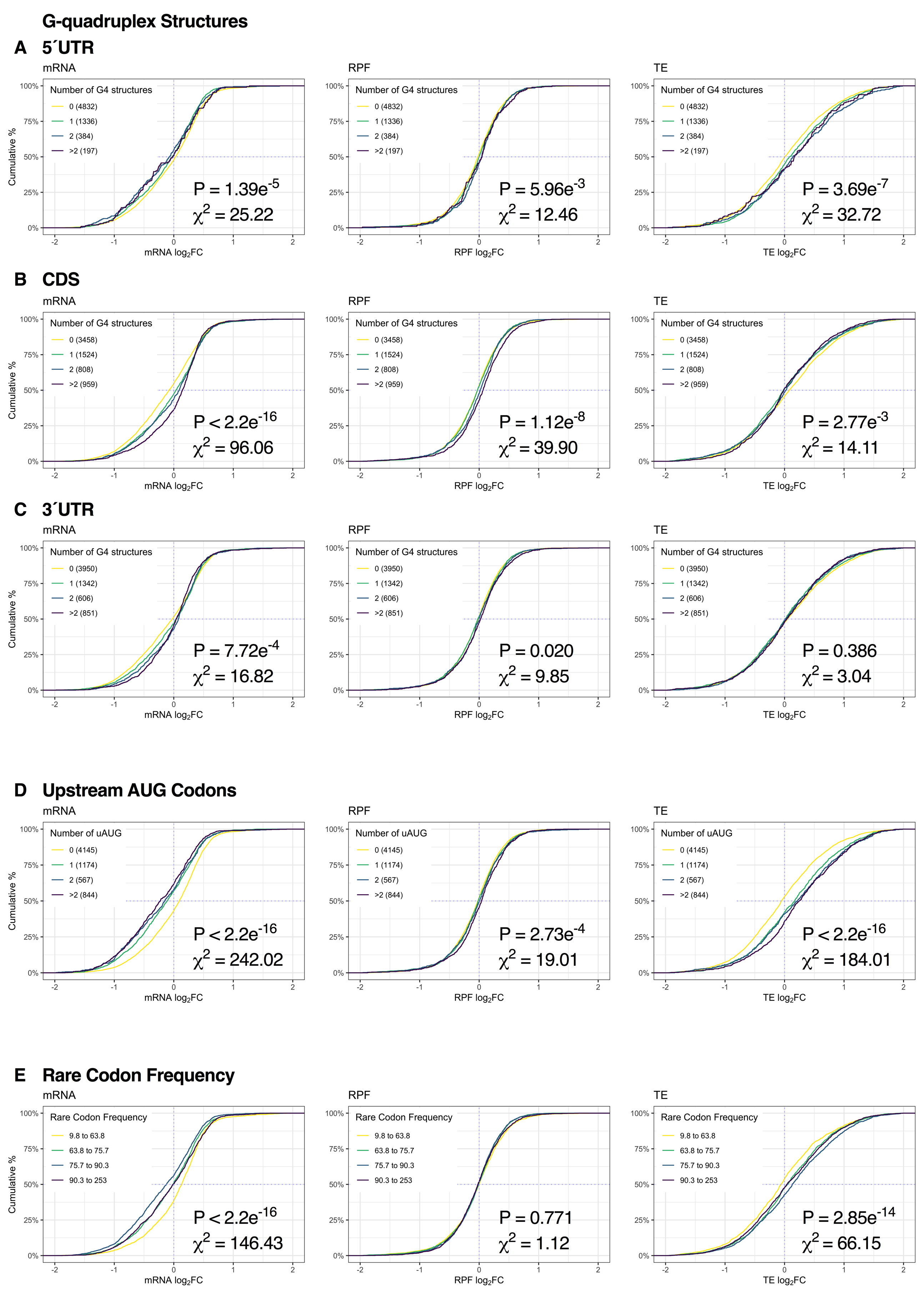
**

**Supplementary Figure 8. Effect of G-quadruplex structures, upstream AUG codons and rare codon frequency on mRNA dynamics immediately after depolarization.** Cumulative density plots depicting changes in mRNA, RPF and TE log_2_FC immediately following depolarisation, after stratification via the number of predicted G-quadruplex structures **(A–C)**, upstream AUG codons **(D)** or rare codon frequency (per 1000 codons) **(E)**. Groups were compared via Kruskal-Wallis test, with associated p-values and 𝜒^2^ test statistics reported bottom right.


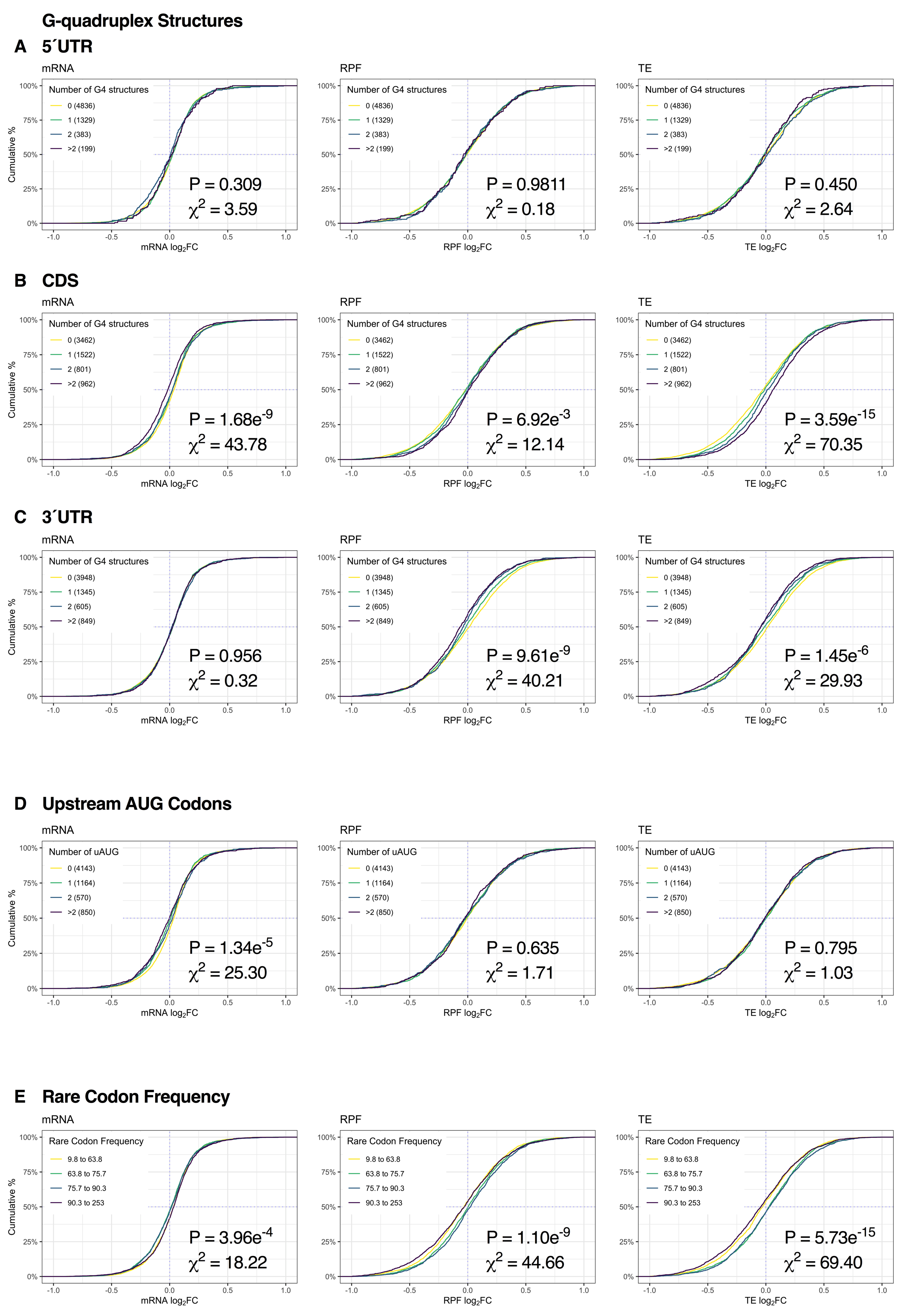


**Supplementary Figure 9. Effect of G-quadruplex structures, upstream AUG codons and rare codons on mRNA dynamics 2 hours after depolarization.** Cumulative density plots depicting changes in mRNA, RPF and TE log_2_FC 2 hours after depolarisation, following stratification via the number of predicted G-quadruplex structures **(A–C)**, upstream AUG codons **(D)** or rare codon frequency (per 1000 codons) **(E)**. Groups were compared via Kruskal-Wallis test, with associated p-values and 𝜒^2^ test statistics reported bottom right.


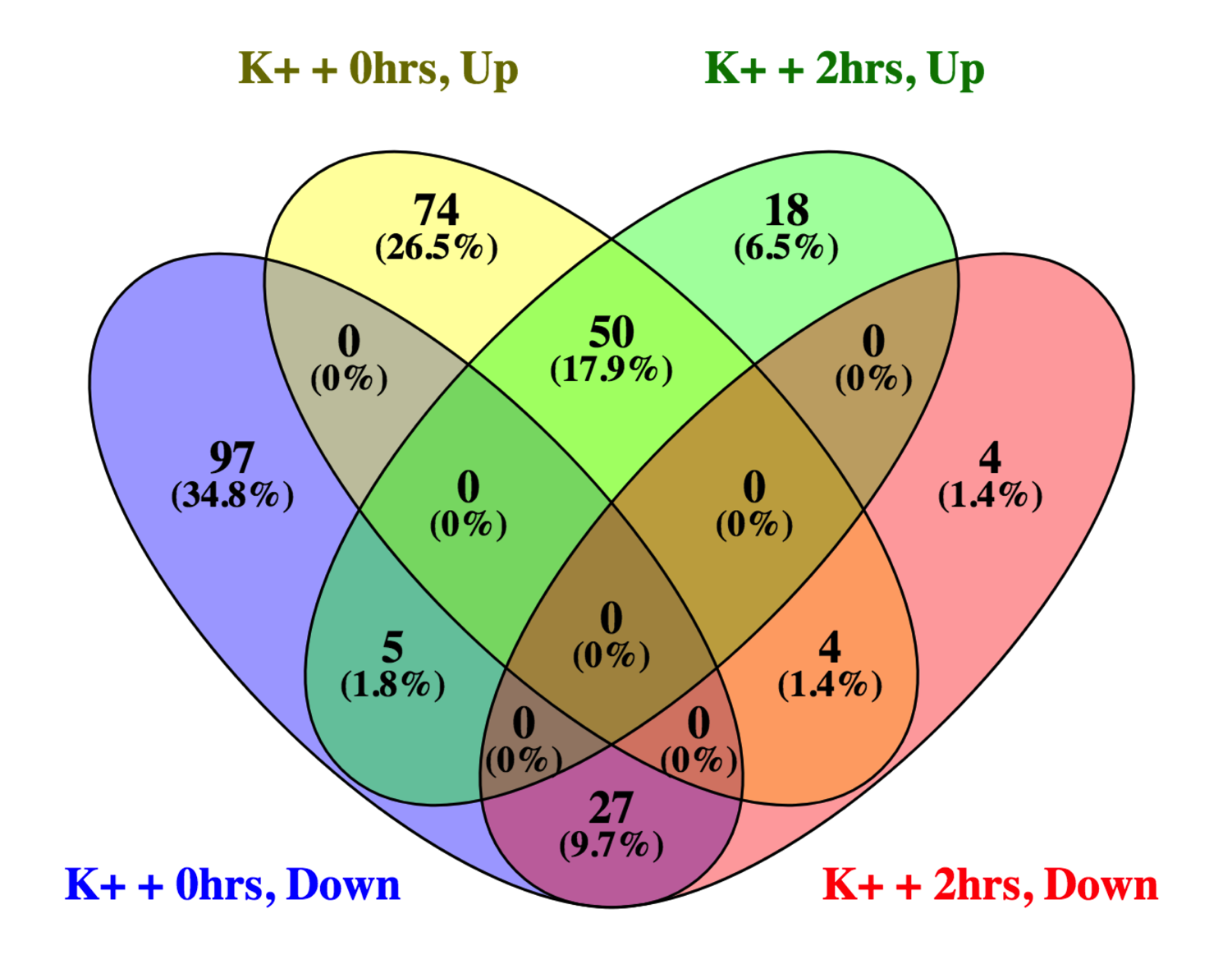


**Supplementary Figure 10.** Venn diagram comparing expression of mature miRNAs immediately and 2 hours after depolarisation.

**
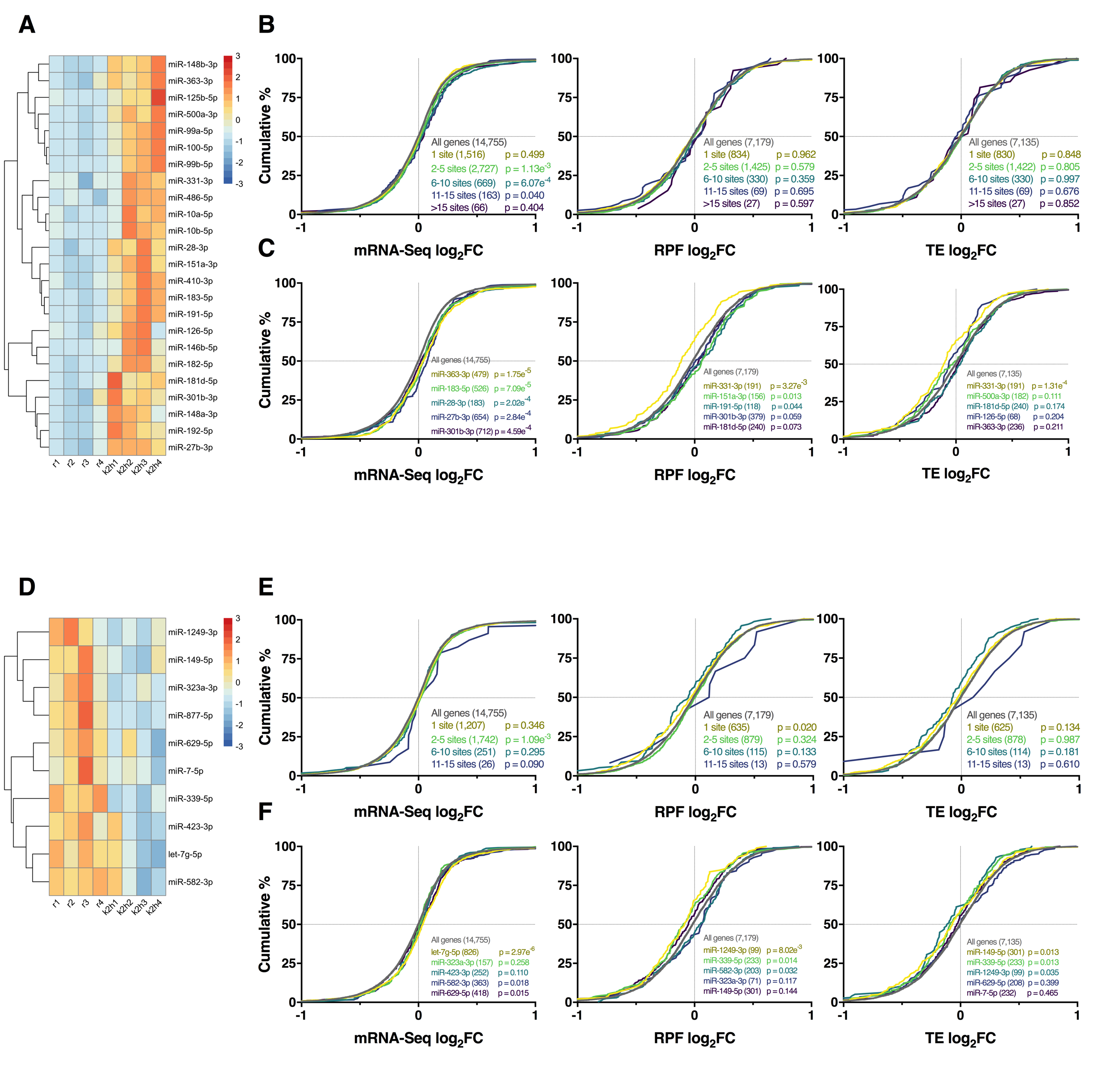
Supplementary Figure 11. MiRNA-mRNA dynamics 2 hours post-depolarisation (A)** Heat map depicting expression of 24 miRNAs both upregulated 2 hours after depolarization and within the top 20% of brain-expressed miRNAs as reported by the miRMine database [1]. Each cell corresponds to the counts-per-million standard deviation relative to the row-wise mean, with red corresponding to high expression, and blue low expression. **(B)** Cumulative distribution profiles of mRNAs collectively targeted by the miRNAs presented in panel **(A)**, stratified by the number of expressed miRNA binding sites. Groups were compared to the entire transcriptome via two-sided Kolmogorov-Smirnov test, with p-values (and numbers of genes analysed) reported bottom right. **(C)** As in **(B)**, except after analyzing the target genes of each miRNA individually. Top 5 miRNAs ranked via p-value are shown. In the case of p-value ties, miRNAs with the largest median difference relative to the entire transcriptome were plotted. **(D)** As in **(A)**, except depicting the expression of 10 downregulated miRNAs within the top 25% of brain-expressed miRNAs. For this analysis, the threshold for brain enrichment was relaxed due to the small number of downregulated miRNAs. **(E & F)** As in **(B & C)**, except after analyzing the target genes of miRNAs depicted in **(D).**
